# Prediction of Protein Binding Regions in Disordered Proteins

**DOI:** 10.1371/journal.pcbi.1000376

**Published:** 2009-05-01

**Authors:** Bálint Mészáros, István Simon, Zsuzsanna Dosztányi

**Affiliations:** Institute of Enzymology, Biological Research Center, Hungarian Academy of Sciences, Budapest, Hungary; University of Bologna, Italy

## Abstract

Many disordered proteins function via binding to a structured partner and undergo a disorder-to-order transition. The coupled folding and binding can confer several functional advantages such as the precise control of binding specificity without increased affinity. Additionally, the inherent flexibility allows the binding site to adopt various conformations and to bind to multiple partners. These features explain the prevalence of such binding elements in signaling and regulatory processes. In this work, we report ANCHOR, a method for the prediction of disordered binding regions. ANCHOR relies on the pairwise energy estimation approach that is the basis of IUPred, a previous general disorder prediction method. In order to predict disordered binding regions, we seek to identify segments that are in disordered regions, cannot form enough favorable intrachain interactions to fold on their own, and are likely to gain stabilizing energy by interacting with a globular protein partner. The performance of ANCHOR was found to be largely independent from the amino acid composition and adopted secondary structure. Longer binding sites generally were predicted to be segmented, in agreement with available experimentally characterized examples. Scanning several hundred proteomes showed that the occurrence of disordered binding sites increased with the complexity of the organisms even compared to disordered regions in general. Furthermore, the length distribution of binding sites was different from disordered protein regions in general and was dominated by shorter segments. These results underline the importance of disordered proteins and protein segments in establishing new binding regions. Due to their specific biophysical properties, disordered binding sites generally carry a robust sequence signal, and this signal is efficiently captured by our method. Through its generality, ANCHOR opens new ways to study the essential functional sites of disordered proteins.

## Author Summary

Intrinsically unstructured/disordered proteins (IUPs/IDPs) do not adopt a stable structure in isolation but exist as a highly flexible ensemble of conformations. Despite the lack of a well-defined structure these proteins carry out important functions. Many IUPs/IDPs function via binding specifically to other macromolecules that involves a disorder-to-order transition. The molecular recognition functions of IUPs/IDPs include regulatory and signaling interactions where binding to multiple partners and high-specificity/low-affinity interactions play a crucial role. Due to their specific functional and structural properties, these binding regions have distinct properties compared to both globular proteins and disordered regions in general. Here, we present a general method to identify disordered binding regions from the amino acid sequence. Our method targets the essential feature of these regions: they behave in a characteristically different manner in isolation than bound to their partner protein. This prediction method allows us to compare the binding properties of short and long binding sites. The evolutionary relationship between the amount of disordered binding regions and general disordered regions in various organisms was also analyzed. Our results suggest that disordered binding regions can be recognized even without taking into account their adopted secondary structure or their specific binding partner.

## Introduction

The classical point of view on protein function claims that the functionality of a protein requires the presence of a well-defined three dimensional structure. However, as the amount of experimental evidence against the generality of this concept grew, this paradigm had to be reassessed [1]. It has become evident that there is a large number of proteins that do not require a stable structure even under physiological conditions in order to fulfill their biological role [2]–[4]. These intrinsically unstructured/disordered proteins (IUPs/IDPs) lack a well defined tertiary structure and exhibit a multitude of conformations that dynamically change over time and population. The importance of protein disorder is underlined by the abundance of partially or fully disordered proteins encoded in higher eukaryotic genomes [5],[6]. Disordered proteins are involved in many important biological functions [2],[7], which complement the functional repertoire of globular proteins [7]. Recent characterization of IUPs based on their functions shows that disorder can help these proteins to fulfill their functions in various ways [8],[9]. In the case of entropic chains, the biological function is directly mediated by disorder (e.g. MAP2 projection domain [10], titin's PEVK domain [11], NF-M and NF-H between neurofilaments [12],[13], nucleoporin complex [14]). Furthermore, disordered segments often act as flexible linkers between folded domains in multidomain proteins [2],[15]. Alternatively, many disordered proteins function by binding specifically to other proteins, DNA or RNA. This process, termed coupled folding and binding involves a transition from disordered state to a more ordered state with stable secondary and tertiary structural elements [16],[17].

The coupled folding and binding confers several functional advantages in certain types of molecular interactions. Since – at least partial – folding happens together with binding, the entropic penalty counterbalances the enthalpy gain coming from the binding [18],[19]. This way disorder uncouples specificity from binding strength allowing for weak transient, still specific interactions that are essential for signaling processes. These properties enable disordered proteins to play an important role in molecular recognition including gene regulation, cell cycle control and other key cellular processes [20]–[23]. The kinetic and thermodynamic details of the binding are influenced by conformational preferences present prior to binding [24]. Although disordered proteins in general lack secondary and tertiary structure, some exhibit partial secondary structure at closer inspection. For example, CD analysis indicated that p21 and p27 possess α-helical segments [19],[25],[26]. Detailed NMR characterization of p27 and other proteins showed that several segments can have a pronounced tendency to adopt α-helical, or even β strand conformations [9]. Upon binding, these inherent structural preferences can either be solidified or overwritten by the partner molecule [27]. Some regions can preserve flexibility even within the complex, mitigating the unfavorable entropy term [28]. This allows the fine-tuning of the affinity of interactions over a wide range. As a general rule, however, these interactions are driven largely enthalpically by the favorable interactions formed with the partner molecule [18],[19],[29].

The inherent flexibility of disordered proteins offers further advantages in binding. It results in a malleable interface that can allow binding to several partners or to adopt different conformations, manifested in increased binding capability [8],[20]. In accordance, several analyses of protein interaction networks revealed that disordered proteins are abundant among hub proteins, proteins with a large number of interacting partners [30],[31]. In a different scenario, the binding partners of an ordered protein are disordered, as shown for binding of 14-3-3 proteins, thus allowing a single protein to bind multiple partners [32]. Beside their involvement in protein-protein interactions, these proteins are also subjects of various post-translational modifications that control their functions, localization and turnover [33]. In this way, these proteins can integrate and mediate multiple signals of various sources, and act as the central elements in signaling or regulatory networks. The centrality of these proteins, however, is also their weakness. It has been suggested that the targeted attack of hubs can cause serious disruption in protein interaction networks [34]. Furthermore, disordered proteins are often associated with various diseases [35]. For example, the primary importance of p53 originates from its involvement of 50% of cancers [36]. In general, 79% of human cancer associated proteins have been classified as IUPs, compared to 47% of all eukaryotic proteins in SwissProt database [22]. Disordered proteins were also suggested to be common in diabetes and cardiovascular diseases [35],[37]. Several disordered proteins - such as Aβ, τ, α synuclein, and prion protein - are involved in neurodegenerative diseases and are also prone to amyloid formation [38]–[40]. On the other hand, due to their specific way of interactions, disordered proteins can also be attractive targets for drug discovery. A novel strategy for drug discovery exploiting binding sites within disordered regions has already been suggested [41]. This adds further support to the importance of finding specific functional sites in proteins that undergo disorder-to-order transition upon binding or *disordered binding regions* in short.

Despite their importance, the number of well characterized examples of disordered proteins undergoing disorder-to-order transition is very small. The PDB also offers only a limited sample of proteins adopting a well defined conformation as part of a complex. However, recent comparisons of these structures with complexes formed between ordered proteins pointed out several differences [42]–[44]. In general, disordered proteins adopted a largely extended conformation in the complex exposing the majority of their residues for interacting with their partner. The interface of disordered proteins was enriched in hydrophobic residues compared to the interface of ordered proteins, but also to disordered regions in general. The higher number of interchain contacts was suggested to be a sign of better adaptation of disordered proteins to the surface of their partner. In general, the regions that become ordered were shorter as compared to globular domains, usually less than 30–40 residues. While the interface of globular proteins was most often formed by distant segments of the amino acid sequence brought together by folding, disordered binding sites were much more localized in the primary structure. These features demonstrate that the underlying principles of molecular recognition of disordered binding regions are different from the complex formation of globular proteins [43].

Disordered binding sites are also expected to be distinguishable from general disordered sites that are not directly involved in binding. A common notion is that protein disorder comes in many flavors, and these should be targeted by specific prediction methods [45],[46]. However, training specific methods would require significantly larger datasets than those that are available today. Nevertheless, existing general protein disorder prediction methods might already be equipped for this problem. It has been suggested that specific patterns of disorder prediction profiles can be associated with regions undergoing disorder-to-order transitions [47]. Since these regions can be ordered as well as disordered, there is no clear recipe whether these regions should be predicted ordered, disordered, or as borderline cases. A recent analysis compared several methods to recognize short protein-protein interaction motifs containing α-helical elements in their bound state, the so-called α-MoRFs [48]. As expected, the various methods showed large variations in predicted order/disorder tendency corresponding to binding regions. One of the earliest prediction method PONDR VL-XT [49]–[51] was quite consistent in predicting these regions as ordered within a broader disordered region, giving them the characteristic appearance of dips in the prediction output. Based on this specific prediction output, a method was developed to recognize α-MoRFs from the amino acid sequence [48],[52]. First, regions predicted with dips in the output of VL-XT were selected and were filtered further by a neural network using several additional properties. This prediction method is restricted to recognize short, α-helical binding regions within disordered proteins.

Here we present a general method to identify specific binding regions undergoing disorder-to-order transition. Our method relies on the general disorder prediction method IUPred [53],[54]. IUPred is based on the assumption that disordered proteins have a specific amino acid composition that does not allow the formation of a stable well-defined structure. The method utilizes statistical potentials that can be used to calculate the pairwise interaction energy from known coordinates. Using a dataset of globular proteins only, a method was developed to estimate the pairwise interaction energy of proteins directly from the amino acid sequence. By virtue of this algorithm, disordered residues can be predicted by having unfavorable estimated pairwise energies. The estimation of the energy for each residue is based on its amino acid type, and the amino acid composition of its sequential neighborhood. Through the amino acid composition of the sequential environment, IUPred can take into account that the disorder tendency of residues can be modulated by their environment [53]. This property of IUPred is exploited in order to recognize regions that are most likely to undergo a disorder-to-order transition based on their estimated pairwise energies in different contexts. The prediction of binding sites is based on estimating the energy content in free and in the bound states, and identifying segments that are potentially sensitive to these changes. In a previous work, the ability to predict specific contacts was emphasized in order to recognize disordered regions that are involved in binding externally rather than internally [46]. In our model, however, there was no attempt made to model specific interactions. Instead, the environment is taken into account simply at the level of amino acid composition. Here we show that this simple model captures the essential property of disordered binding regions and allows their robust prediction. We termed our disordered binding site prediction method ANCHOR, to reflect the primary importance of short segments driving the complex formation between a disordered protein and its partner.

## Results

### The outline of the algorithm

The goal of the present work was to recognize a special class of disordered segments from the amino acid sequence, namely those that are capable of undergoing a disorder-to-order transition upon binding to a globular protein partner. The essential feature of such binding regions is that they behave in a characteristically different manner in isolation than bound to their partner protein. In their free state, they behave as disordered proteins, existing as a highly flexible structural ensemble. In their bound state they usually adopt a rigid conformation, similar to regions within globular structures. This capability to behave in drastically different ways in different environments is targeted by our approach. We seek to identify segments in a generally disordered region that cannot form enough favorable intrachain interactions, however they have the capability to energetically gain by interacting with a globular partner protein. Our prediction is based on three properties.

1. The first criterion ensures that a given residue belongs to a long disordered region, and filters out globular domains.
2. The second criterion corresponds to the isolated state and it ensures that a residue is not able to form enough favorable contacts with its own local sequential neighbors to fold, otherwise it would be prone to adopt a well defined structure on its own.
3. The third criterion tests the feasibility that a given residue can form enough favorable interactions with globular proteins upon binding. This basically ensures that there is an energy gain by interacting with globular regions.

These properties are estimated individually and are combined into a single predictor via optimized weights.

In more detail, the prediction of these three properties relies on the energy estimation framework implemented in IUPred, a general disorder prediction method. The core element of IUPred is the energy predictor matrix **P**. The parameters in *P_ij_* were trained on globular proteins with known structures only, without relying on any kind of disordered dataset. These parameters were determined to minimize the difference between the estimated energies and the energies calculated from the known structures on the dataset of globular proteins. Using the energy predictor matrix IUPred predicts the *E* interaction energy for each residue based on the following formula in default:
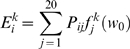
(1)where *i* denotes the type of the *k*-th amino acid, *P_ij_* is the element of the energy predictor matrix that estimates the pairwise energy of residue of type *i* in the presence of residue type *j*, 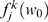 is the fraction of residue type *j* in the sequential environment within *w_0_* residues from residue *k*. The size of neighborhood considered (*w_0_*) equals 100 residues in both directions and the result is smoothed over a window size of 10 (also in both directions from the *k*-th residue so in fact 21 residues are considered in total). For the final prediction output, the energies are transformed into probability values, denoted as *s_k_*. For more details see Dosztányi et al. [53].

The disordered binding site prediction is based on three different scores that are calculated with a slight modification of the original energy estimation scheme. The parameters of *P_ij_* were taken directly from IUPred. The following three scores are assigned to each residue in a protein according to the above described criteria (1–3):

1, To measure the tendency of the neighborhood of an amino acid for being disordered we use the IUPred algorithm and assign an *S_k_* score to the *k*-th residue of the chain by averaging the IUPred scores in the *w_1_* neighborhood of the residue in question:
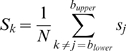
(2)where *s_j_* is the IUPred score of the *j*-th residue of the chain, N is the number of amino acids in the averaging and *b_lower_* and *b_upper_* are the lower and upper boundaries of the neighborhood of the *i*-th residue, that is *b_lower_* = max(*k*−*w_1_*;1) and *b_upper_* = min(*k+w_1_*;*l*), where *l* is the chain length.

2, We estimate the pairwise interaction energy the given residue may gain by forming intrachain contacts. This is done the exact same way as in IUPred using (1), only here the size of the considered neighborhood (*w_2_*) is left as a parameter and is set during the training of the predictor:
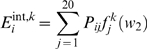
(3)The smaller window size corresponds to more local behavior.

3, The pairwise energy that the residue may gain by interacting with a globular protein is approximated using the average amino acid composition of globular proteins:
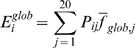
(4)where 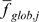 is the fraction of residue type *j* in the averaged reference amino acid composition of globular proteins shown in Table 1. By subtracting this energy from 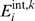 one can estimate the energy that the residue may gain by interacting with a hypothetical globular protein compared to forming intrachain contacts (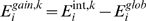).

**Table 1:** Reference amino acid composition of globular proteins.

*AA*	*F* %
R	3.68
K	6.37
D	4.92
E	5.43
N	4.69
Q	3.86
S	8.05
G	8.46
H	2.00
T	6.35
A	7.67
P	4.89
Y	3.86
V	7.13
M	1.84
C	2.43
L	8.22
F	3.19
I	5.20
W	1.76

Amino acid composition of the reference globular protein dataset comprised of all the amino acids in the longer chains of the ordered complexes dataset. Amino acids are sorted by increasing hydrophobicity based on the Fauchere-Pliska hydrophobicity scale [94]. *AA* denotes amino acid codes and *f* denotes the fraction of the respective amino acid expressed as a percentage.

The final prediction score of the residue is given by the linear combination of the above three terms:
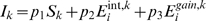
(5)where the *p_1_*, *p_2_* and *p_3_* coefficients are determined during the training of the predictor together with the optimal values of *w_1_* and *w_2_* window sizes. *I_k_* is then converted into a *p* value that expresses the probability of that residue being in a disordered binding site. For a binary classification residues with scores above 0.5 are predicted to be in a disordered binding site. Since the second and third terms of (5) may vary heavily between neighboring residues, the final score is smoothed in a window of 4 residues.

The optimal values for the three weights (*p_1_*, *p_2_* and *p_3_*) and the two window sizes (*w_1_* and *w_2_*) are determined using a dataset of disordered protein complexes and ordered monomeric proteins by three-fold cross validation (See Methods and Figure S1 for a schematic representation and outline of this procedure). The small dataset of known disordered proteins bound to ordered proteins represent a serious bottleneck during optimization. Therefore, it is a clear advantage of our approach that it greatly reduces the dependence on the existing dataset of disordered complexes, and leaves us with only 5 parameters to be optimized on this small dataset.

**Figure 1:**
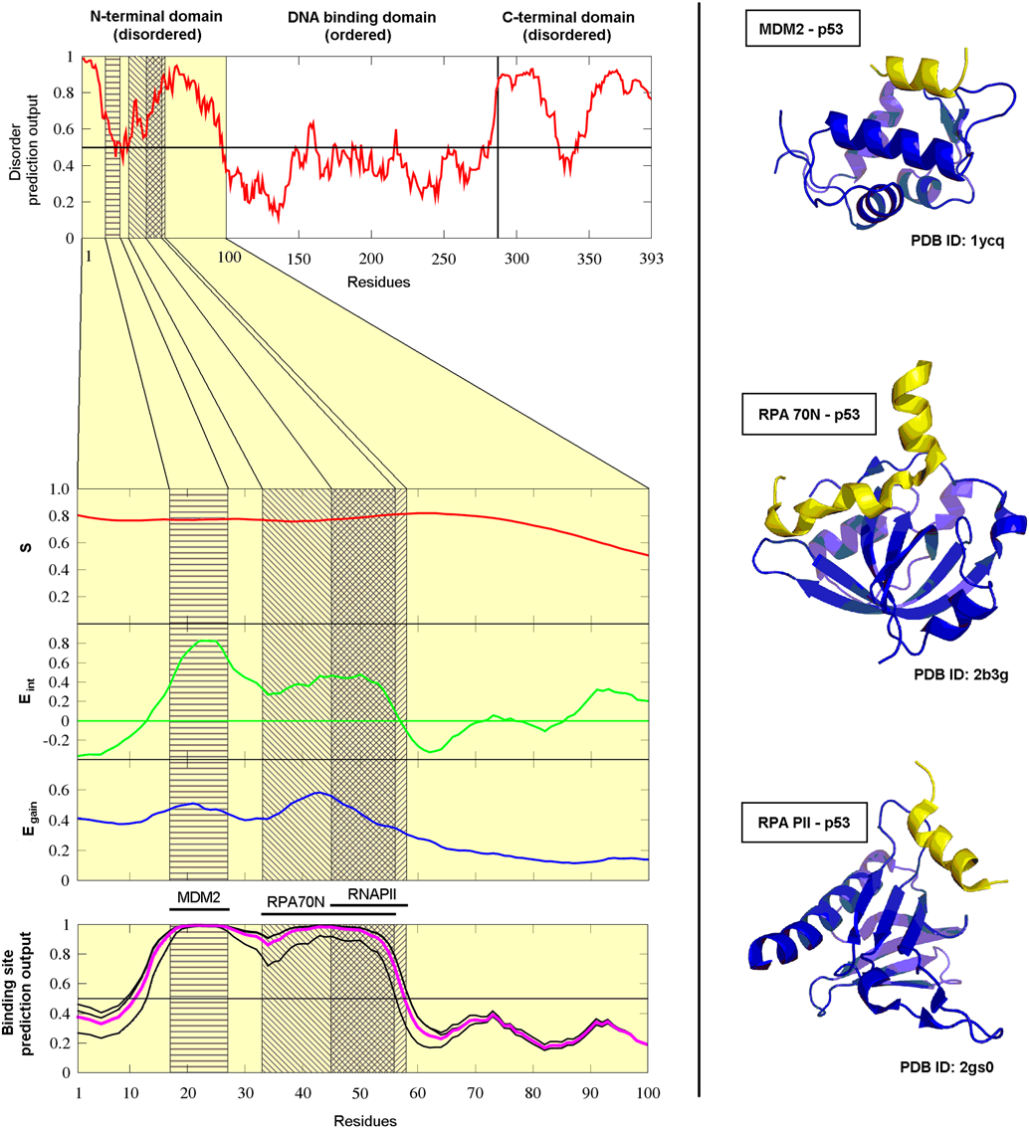
The construction of the ANCHOR prediction method demonstrated on the N-terminal domain of human p53. *Left:* IUPred prediction score for the full length human p53 (top) and *S*, *E_int_* and *E_gain_* calculated for the disordered N terminal domain of human p53 (middle). Grey boxes show the three binding sites with the overlap of the RPA70N and RNAPII binding sites shown in dark grey. The outputs of the three individually optimized predictors are shown in black and their average, the final prediction score is shown in purple (bottom). *Right:* PDB structures of the binding sites in the N-terminal region of p53 (yellow) complexed with the respective partners (blue): MDM2 (top, PDB ID: 1ycq [57]), RPA 70N (middle, PDB ID: 2b3g [58]) and RNA PII (bottom, PDB ID: 2gs0 [59]).

The behavior of various scores is shown for an example, the N terminal domain (residues 1–100) of human p53 tumor supressor protein that plays an important regulatory role [55]. Its N terminal region is completely disordered [56] and is known to be able to bind to (at least) three different globular proteins as shown in Figure 1. The segment between residues 17–27 binds to MDM2 [57], the other two binding sites overlap with residues 33–56 binding to RPA 70N [58] and residues 45–58 binding to the B subunit of RNA polymerase II [59]. The three calculated quantities for this domain are also shown in Figure 1. It is worth noting that the MDM2 binding site in the N-terminal region of p53 appears to be on the border of being disordered. Although the disordered prediction is part of ANCHOR, the output of this prediction (*E_int_*, described in Theory) is linearly combined with two other quantities meaning that predicted disorder is not strictly a prerequisite of a successful disordered binding site prediction.

### Testing of the algorithm

Testing of the predictor was done by dividing both our negative and positive datasets (*Globular proteins* and *Short disordered complexes*) into three subsets, training the predictor on two of these and evaluating it on the remaining third one. This was done in all three possible combinations yielding three optimal parameter sets. The parameters calculated on the training sets are shown in Table 2 together with the respective True Positive Rates (TPR) and the fraction of the amino acids in disordered regions of the Disprot dataset predicted to be in disordered binding sites (F values). The optimal parameters were chosen to maximize the amount of correctly predicted disordered binding sites (TPR) while minimizing predicted binding sites in globular proteins (FPR) and also restricting predicted binding sites within disordered regions in general (F). The fact that the three parameter sets do not differ significantly implies that our method is robust.

**Table 2:** Parameter and prediction accuracy values obtained during the optimization of ANCHOR.

	*w_1_*	*w_2_*	*p_1_*	*p_2_*	*p_3_*	*F* (%)	*TPR* (%)	*FPR* (%)
Training set 1	25	60	0.4630	0.3847	0.7985	46.0	69.8	5.0
Training set 2	27	60	0.6075	0.4149	0.6773	47.4	67.7	5.0
Training set 3	29	90	0.6990	0.4585	0.5488	43.4	64.8	5.0

Optimal parameters of the predictor determined during training. *w_1_*, *w_2_*, *p_1_*, *p_2_* and *p_3_* are the optimized parameters, *F* is the fraction of the residues in the disordered regions in the Disprot database that are predicted to be in binding sites, *TRP* and *FPR* are the True- and False Positive Rates, respectively.

The output of the predictor with all three parameter sets and the combined final predictor (the average of these three) are shown for the example of the N terminal region of p53 in Figure 1. A few additional well characterized examples are shown in the Supporting Information (Figure S2, Figure S3, Figure S4, Figure S5, and Figure S6).

The results obtained on the three independent testing subsets as well as their average are given in Table 3. Since the cutoffs are given by the training process such that we achieve exactly 5% False Positive Rate (FPR) on the respective training sets (ie. the part of the original Globular proteins dataset that was used in the training of the respective subpredictor), the FPR's are also quoted (they can differ slightly from 5%). Besides the overall TPR calculated on a residue basis (marked *TPR_AA_*), we also calculated the percentage of binding sites identified, termed *TPR_SEG_*. A binding site was considered to be found if at least five of its amino acids are correctly classified. The results show that ANCHOR performs at 62% *TPR_AA_* with a slightly higher *TPR_SEG_* of 68% on average, while maintaining a 5% FPR. ANCHOR is also specific to disordered binding sites as opposed to disorder to general. If all disordered proteins had approximately equal capability of binding then the fraction of correctly identified disordered binding sites (TPR) could not be significantly different from the fraction of disordered regions predicted to be binding sites (F value). As this is not the case (TPR = 62% vs. F = 42%) we can conclude that common features of known disordered binding sites that distinguish them from general disordered protein regions are successfully recognized.

**Table 3 pcbi-1000376-t003:** Prediction efficiency of ANCHOR evaluated on the testing datasets.

	*TPR_AA_* (%)	*TPR_SEG_* (%)	*FPR* (%)
Testing set 1	61.1	62.5	5.7
Testing set 2	69.5	80.0	4.4
Testing set 3	54.7	62.5	5.1
Average	61.8	68.3	5.1

Results of the testing of ANCHOR on the three testing datasets. *TPR_AA_* denotes the ratio of correctly identified amino acids belonging to binding sites. *TPR_SEG_* denotes the ratio of binding sites found by the algorithm.

**Figure 2:**
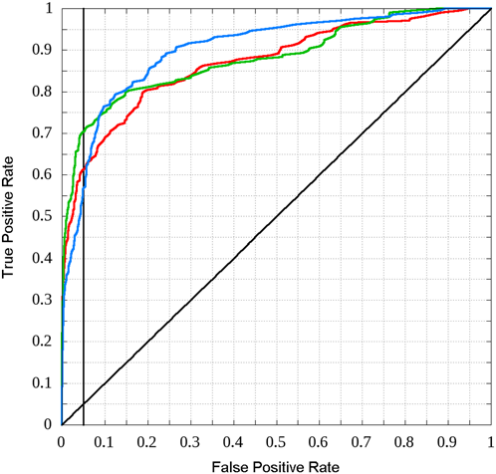
ROC curves obtained during the testing of ANCHOR. ROC curves of the predictor with parameter sets optimized on each of the three training subsets and evaluated on the respective testing subsets are shown with red, green and blue lines. The line with unity slope corresponding to random prediction is also shown. The vertical line corresponds to FPR = 0.05, where the final predictor (the average of these three) is used.

Another standard way of describing prediction algorithms is by Receiver Operating Characteristic (ROC) curves [60], that is the TPR versus the FPR of the algorithm. This relationship is mapped by scanning the interval between 0 and 1 with the score cutoff. The three ROC curves of the predictor with the three different parameter sets evaluated on the respective testing sets are shown in Figure 2. A single number measure to characterize the performance is the area under the curve (AUC) with random predictors scoring AUC = 0.5 and perfect predictors scoring AUC = 1. The AUC values of the predictors trained and tested on the respective subsets are 0.8675, 0.8781 and 0.8993.

Since the interacting regions of a disordered and an ordered protein are inherently different we expect that the predictor will only recognize binding sites in disordered proteins that interact with globular proteins but are not part of globular proteins themselves. In order to verify this hypothesis we tested the combined final predictor on a dataset of complexes containing only ordered chains (that is three-state complexes – see Methods). The prediction was done on the short interacting chain of the complexes. This gave a false positive rate of only 3.7% that is even lower than the value obtained on our testing set, although this might be only a consequence of the relatively small size of our ordered complex set (72 complexes). Overall, we could ensure that our predictor makes very few mistakes on both globular proteins and complexes of globular proteins, while it can still recognize the majority of disordered binding regions. This implies that our algorithm is specific to disordered binding sites as opposed to globular proteins, the interface between globular proteins or disordered proteins in general.

Our predictor was also tested on a completely independent dataset of *α-MoRFs*, short disordered complexes that was assembled by Cheng et al. [48] and composed of 40 proteins containing binding regions that adopt mostly α-helical structure upon binding. The results of the prediction on this dataset can be seen in Table 4. Although the residue based TPR is somewhat lower than that calculated on our testing set (57.0% instead of 61.8%), the segment based TPR is almost the same for the two sets (67.5% and 68.3%). Overall these results are comparable to the ones calculated on our training set.

**Table 4:** Prediction efficiency of ANCHOR evaluated on an independent dataset (α-MoRFs dataset).

	H	E	C	Total	SEG
In dataset	263	8	210	479	40
Found	147	5	121	273	27
Ratio (TPR)	55.9%	62.5%	57.6%	57.0%	67.5%

Prediction results for the α-MoRFs dataset. SEG denotes segment based results where each binding site is considered one segment and one such segment is considered found if at least five of its amino acids are correctly identified.

### Amino acid based evaluation of the predictor

**Figure 3:**
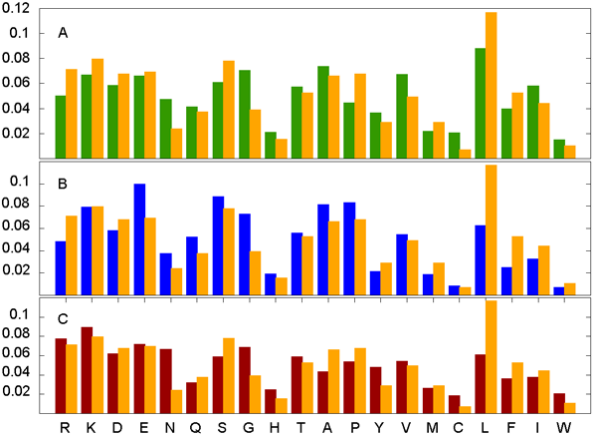
The distinct amino acid composition of short disordered binding sites. The average amino acid composition of the interacting parts of the short disordered binding sites compared to the average amino acid composition of (A) the globular proteins dataset, (B) the disordered proteins dataset and (C) the interacting parts of the shorter chains of the ordered complexes. Amino acids are arranged according to increasing hydrophobicity.

The specific construction of the algorithm for the prediction of interaction energy implies that the method will be sensitive to amino acid compositions. The differences between the composition of disordered binding sites and the amino acid composition of any of the negative sets (globular proteins, ordered interfaces and disordered proteins in general) are shown in Figure 3A, 3B, and 3C, respectively. The amino acid compositions of all three datasets are significantly different from that of disordered binding segments (data not shown).

The final prediction is based on three different scores that combine local and global disorder tendency with sensitivity to the structural environment. Although the individual quantities that are combined for the final score can work selectively better or worse for various types of residues, the effect of these differences on the efficiency of the final prediction is not trivial. This effect was tested by comparing the amount of the different amino acids in the short disordered binding sites to the amount recovered from these by the predictor. These data are shown in Table 5 together with the calculated *p* values quantifying their differences. As all of the *p* values are fairly large, these differences are likely to occur by chance alone. For example, proline rich binding sites are found with similar accuracy as binding sites enriched in hydrophobic amino acids. Therefore, one may conclude that there is no statistical evidence based on the available dataset that the efficiency of the predictor depends significantly on the amino acid composition of the disordered binding site in question.

**Table 5:** The independence of the efficiency of ANCHOR from the amino acid composition of the binding sites.

*AA*	*N_int_*	*N_found_*	*p*
R	42	21	0.122
K	47	36	0.362
D	40	27	1.000
E	41	20	0.116
N	14	6	0.252
Q	22	11	0.358
S	46	34	0.497
G	23	14	0.758
H	9	7	1.000
T	31	20	1.000
A	39	33	0.068
P	40	19	0.113
Y	17	11	1.000
V	29	20	1.000
M	17	16	0.085
C	4	2	1.000
L	69	47	0.857
F	26	19	0.764
I	31	26	0.146
W	6	5	1.000

*N_int_* shows the number of interacting residues in the short disordered binding sites, *N_found_* shows the amount of these that are correctly found by the predictor. As there are types of amino acids that are rare, Fisher's exact test was used to calculate (two-tailed) *p* values to determine if the predictor works significantly better or worse for certain amino acid types with high p values corresponding to no significant difference.

### Secondary structures and the efficiency of ANCHOR

**Figure 4:**
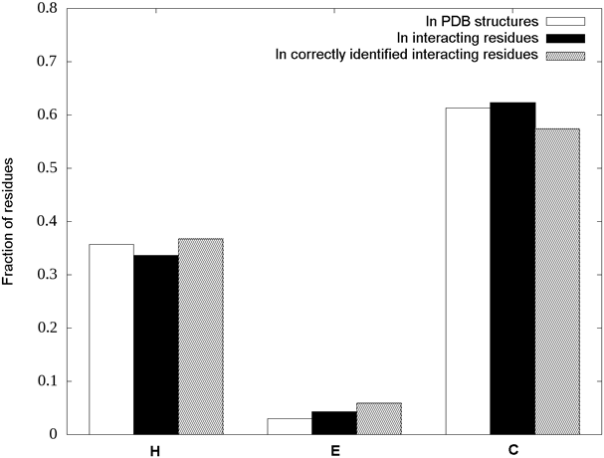
Secondary structure distributions in the short disordered binding site dataset. Fraction of amino acids in different secondary structures in the disordered chains of the complexes. The three groups denote the fractions calculated on all the residues in the PDB structures, only the interacting ones and the ones correctly identified by the predictor.

The relationship between the efficiency of the prediction and the secondary structure types was also assessed, by considering the three types of secondary structural elements: helix (H, including α- and 3_10_ helices), extended (E) and coil (C, including everything else) as defined by DSSP [61]. The number of amino acids in different conformations that can be found in the PDB structures of our positive training set (short disordered complexes), in the interacting residues of these structures and the interacting residues that are correctly identified by the predictor are shown in Table 6. These data are represented graphically as distributions in Figure 4. The secondary structure content in this type of interactions is heavily biased towards coil conformation. It can also be seen on Figure 4 that the predictor seems to work slightly better for H and E conformations. However assessing the difference of the distributions of secondary structures in interacting residues and in the subset identified correctly by ANCHOR shows that this difference is not statistically significant at a 5% level (χ^2^ = 5.32, *p* = 0.070).

**Table 6:** Secondary structure distributions in the short disordered binding site dataset.

	Total in PDB		Interacting residues		Correctly identified	
	Number	Fraction (%)	Number	Fraction (%)	Number	Fraction (%)
H	297	35.7	200	33.6	144	36.7
E	25	3.0	25	4.2	23	5.9
C	510	61.3	371	62.2	225	57.4
**Total**	**832**		**596**		**392**	

The number and fraction of amino acids in different secondary structures in the disordered chains of the complexes. The three groups show these data for all the amino acids in the PDB structures, the ones in interaction and the ones that are correctly identified as part of binding site by ANCHOR.

Furthermore, a similar result holds true if binding sites are categorized based on their dominant secondary structure type - that is there is no significant correlation between the secondary structure type the binding regions adopt upon binding and the efficiency of the predictor. (Dataset S1 shows the secondary structure types determined for the short disordered chains in the disordered complexes as described in Protocol S1.) Overall, this means that there is no significant difference in the efficiency of the prediction on different secondary structural elements.

### Testing on long disordered regions

**Figure 5:**
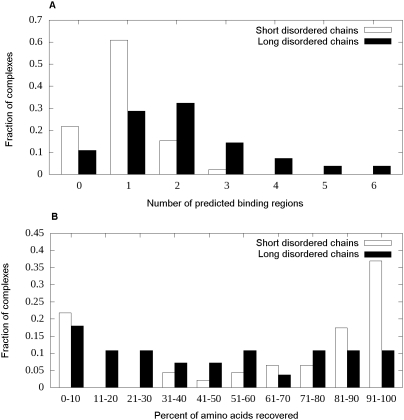
Prediction accuracies and segmentation for the short and long disordered binding sites. (A) The distribution of the number of binding segments predicted in short (white bars) and long (black bars) binding sites. It shows the segmented nature of longer binding sites. (B) The distribution of the fraction of correctly recovered interacting residues in both the short (white bars) and long (black bars) disordered binding sites.

Since the predictor was trained on the short disordered dataset it is informative to see how it performs on long disordered binding sites. There is experimental evidence that at least some long disordered chains are not uniform concerning binding strength but contain short stretches of strongly interacting residues separated by segments that interact with the partner only weakly if at all [19]. In these cases, it is expected that the predictor will be unable to identify the weakly interacting parts since – though these parts may also form interchain contacts – they would not be able to bind to the partner in the absence of their sequential neighbors. The distribution of predicted binding regions for the short and long disordered chains in Figure 5A shows a strong preference for predicting multiple interacting regions for longer chains. This inevitably yields lower residue based TPR but the segment based TPR is not expected to drop. Testing the predictor on the long disordered data confirms this assumption with a decreased residue based TPR of 47.7% (as opposed to 65.8% obtained on running the final predictor on the whole set of short disordered complexes) but with a basically unchanged segment based TPR of 78.6% (compared to the 76.1% calculated on short disordered complexes). These data suggest that the method either finds short disordered binding sites as a whole or completely misses it. However, this may not be true for long binding regions. Figure 5B shows the distribution of the fraction of amino acids successfully identified during prediction in the two types of binding sites. The effect can clearly be seen as about 59% of short binding regions are either fully recovered or are completely missed (the sum of the rightmost and leftmost columns) whereas this ratio is only about 29% for long binding sites.

This type of behavior is illustrated on the disordered human p27. This protein is involved in controlling eukaryotic cell division through interactions with cyclin-dependent kinases. Its kinase inhibitory domain binds both subunits of the CDK2-cyclin A complex in an extended conformation (PDB ID: 1jsu [62]). It is known from kinetic measurements that the binding of p27 is hierarchical through its three domains: first, the D1 domain (residues 25–36) binds to cyclinA which anchors the neighboring LH domain (residues 38–60) that exhibits transient helical structure in monomer state as well [63]. After the binding of D1 this transient structure is stabilized and positions the rest of the chain (D2 domain, residues 62–90) in the correct position to bind to CDK2.

**Figure 6:**
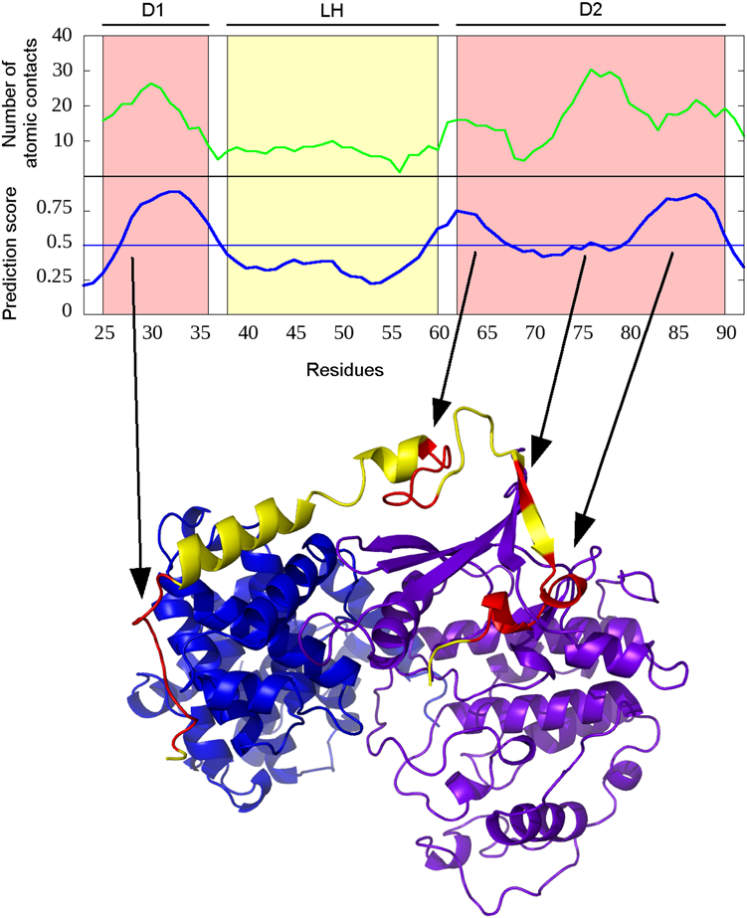
ANCHOR prediction for human p27. *Top:* Number of atomic contacts (green) and prediction output (blue) and for the N-terminal binding region of human p27. “D1”and “D2” denote the two strongly interacting domains (red boxes) and “LH” denotes the weakly interacting linker domain between them (yellow box). *Bottom:* Crystal structure of human p27 (red and yellow) complexed with CDK2 (magenta) and Cyclin A (blue) (PDB ID: 1jsu [62]). Red parts denote regions that are predicted to bind by the predictor. These regions correspond to the experimentally verified strongly binding regions of p27. The figure was generated by PyMOL.

Figure 6 shows the prediction output for p27. Four interacting regions are identified with the first one (27–37) clearly corresponding to D1. The gap between the first two regions (38–58) coincides with the weakly interacting LH domain. The last three regions (59–67, 74–77 and 79–90) cover the strongly interacting D2. Figure 6 also shows the number of atomic contacts/residue for p27 (averaged in a window of size 3). This contact number profile exhibits well pronounced peaks that line up with the regions that are predicted by our algorithm. The figure also shows the four predicted regions mapped to the crystal structure of the complex.

### Wiskott-Aldrich Syndrome protein (WASp)

The examples discussed so far represent various fragments of proteins. Here we present an additional case showing the prediction output for a complete protein sequence.

**Figure 7:**
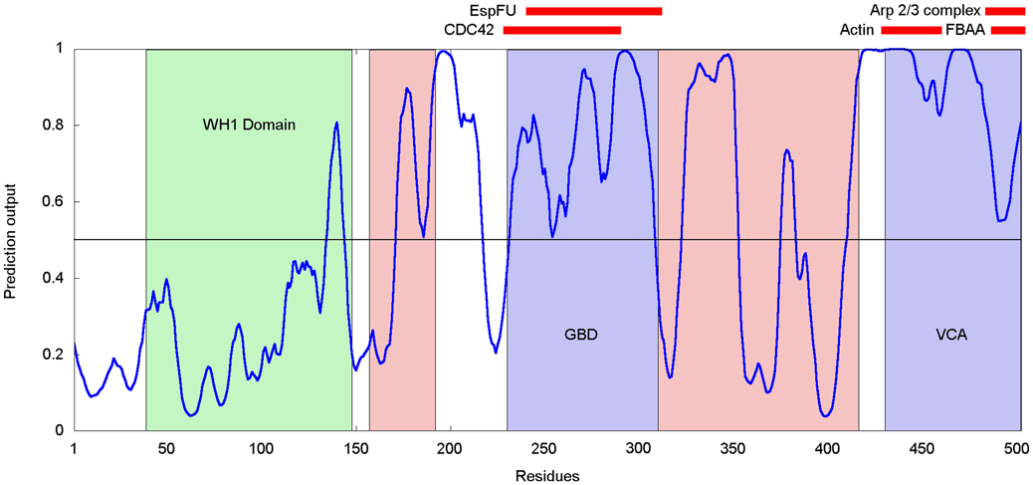
ANCHOR prediction for human WASp. Red bars mark known interaction sites, green box marks the globular WH1 domain, blue boxes mark the GBD and VCA domains. Light red boxes indicate the regions with putative SH3 domain interaction sites.

The human Wiskott-Aldrich Syndrome protein (WASp) is a 502 residue long protein that is expressed in the cells of the hematopoietic system [64]. Its mutations can be linked to the Wiskott-Aldrich Syndrome (WAS), a disease characterized by actin cytoskeleton defects leading to deficiencies in blood clotting and immune response. The protein is composed of various functional domains. It contains the WH1 domain near the N terminus (residues 39–148), the GTPase-binding domain (GBD, 230–310), a polyproline-rich region and a C-terminal verpolin homology/central region/acidic region (VCA, 430–502) domain [65] that also contains the WH2 domain (430–447). Apart from the structured WH1 domain, it is predicted to be largely disordered and contains several low complexity regions (enriched in P, G and acidic amino acids). There is experimental evidence that the activated WASp hubs a number of interactions with partners including CDC42, RAC, NCK, FYN, SRC kinase FGR, BTK, ABL, PSTPIP1, WIP, and the p85 subunit of PLC-gamma as well as the Arp2/3 complex. However, the location of many of these binding regions is not known. The domain structure of WASp is shown in Figure 7 together with the known binding regions.

In its inactive state WASp exists in an autoinhibited form with the GBD domain bound to the VCA domain. When WASp is activated, the GBD domain is bound to CDC42 and this interaction disrupts the GBD-VCA interaction. This initiates a conformational change where WASp opens up and becomes able to bind to the Arp2/3 complex leading to its activation and actin nucleation. Both GBD and VCA regions were shown to be disordered in their free state [65],[66], with GBD adopting a loosely packed, compact conformation. However, the structure of both complexes could be determined using NMR, by covalently linking GBD to CDC42 or the VCA region, respectively [65],[67]. In these two structures WASp GBD adopts related but distinct folds. The plasticity that can be seen by comparing these two complexes is enabled by the absence of discrete tertiary structure in isolation. As it can be seen on Figure 7, ANCHOR captures these disordered binding sites correctly.

It is known that WASp is able to bind to SRC Homology 3 (SH3) domains through one of its proline rich regions although the exact binding site is not known. The interaction with SH3 domains is usually mediated by a short, linear sequence motif that is present in the interaction partner. In the collection of Eukaryotic Linear Motifs (ELM) database (http://elm.eu.org/ [68]) there are five different motifs annotated as SH3 recognition sites. Multiple instances of the following three can be found in human WASp: LIG_SH3_1, LIG_SH3_2 and LIG_SH3_3 represented by the following consensus sequences: [RKY]..P..P, P..P.[KR] and …[PV]..P, for interaction with Class I/ClassII SH3 domains and those SH3 domains with a non-canonical Class I recognition specificity, respectively. The found motifs are clustered in two separate regions mainly falling into the proline-rich regions of WASp (Figure 7). Although there is no direct evidence for the location of interaction with SH3 domains on human WASp, the interaction sites have been identified for Las17 [69], the yeast homologue of this protein. In total, four distinct regions containing multiple binding sites were identified experimentally in Las17 that interact with various SH3 domains. These sites correspond to the proline rich regions in WASp (155–194 and 306–427) that also match with several SH3 binding motifs. As linear motifs were shown to have a preference to reside in disordered regions [70], it is plausible to expect ANCHOR to be able to recognize the SH3 binding region of WASp. In accordance with this, both regions containing putative SH3 binding sites contain binding sites predicted by ANCHOR. This prediction can restrict the candidate sequence regions for SH3 binding and can guide experimental studies to localize true binding sites.

### Complete proteome scans

**Figure 8:**
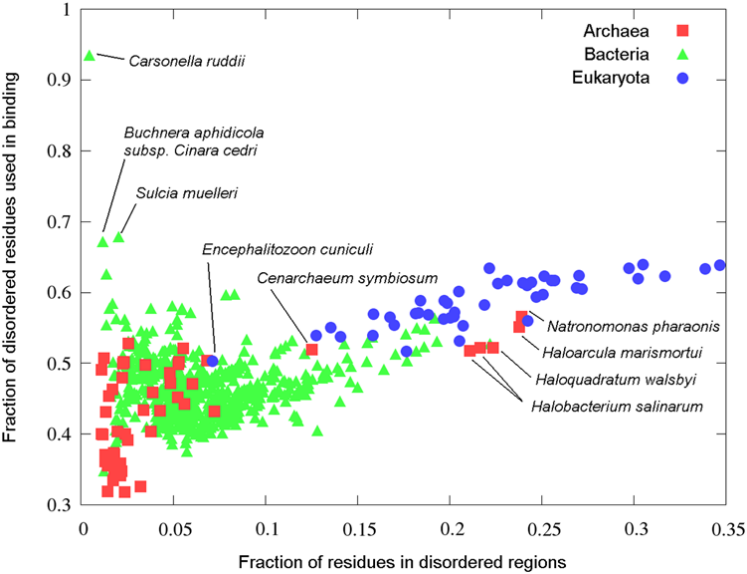
Fraction of disordered and disordered binding site residues in complete proteomes. The number of amino acids in disordered binding sites divided by the number of amino acids in disordered regions plotted as a function of the number of amino acids in disordered regions divided by the total number of residues in the proteome of the organism for the 736 complete proteomes deposited in the SwissProt database, colored according to the three kingdoms of life. The outlying points are marked with the name of the corresponding organism.

In order to gain some evolutionary insight concerning disordered binding sites, the predictor was run on the 736 complete proteomes (53 archaea, 639 bacteria and 44 eukaryota, see Dataset S5, Dataset S6, and Dataset S7, respectively) that are currently available from the SwissProt database (ftp://ftp.expasy.org/). In agreement of previous analyses [5],[6] there is a clear trend of increasing amount of protein disorder as the complexity of the organism increases (see Figure 8). However, Figure 8 also shows that the fraction of disordered amino acids predicted to be in disordered binding sites increases even compared to fraction of disordered residues, as the complexity of organisms grows. Generally, archaea have the least amount of both disorder and binding sites. On the other hand, eukaryota have generally the largest ratio of disordered and binding amino acids with bacteria being between these two groups on average. However there are a few exceptions to these general trends, marked separately on Figure 8.

Considering archaea, mesophiles generally contain a larger amount of disorder and a larger fraction of disordered binding sites than most extremophiles (thermophiles, cryophiles and acidiphiles). However the group of halophile archaea (archaea that favor high saline concentration) is a distinct exception with fraction of disordered amino acids ranging from 0.2 to 0.25 as opposed to other extremophiles' values not exceeding 0.07. This group includes all the halophile archaea in our study, namely *Natronomonas pharaonis*, *Haloarcula marismortui*, *Haloquadratum walsbyi* and two types of *Halobacterium salinarum*. *Cenarchaeum symbiosum*, the only example of obligate endosymbiont among archaea also has an unusually large amount of disordered protein segments in its proteome (0.12). While *Cenarchaeum symbiosum* is closely related to thermophile archaeas, it is adopted to the much lower living temperature of its host [71]. This adaptation could explain the relatively large amount protein disorder and disordered binding sites. In general, these clear differences in the predicted disorder between various archaea organisms points to different strategies to adapt to various extreme environmental conditions resulting in biased amino acid compositions. However, we cannot rule out the possibility that under such extreme conditions, as high salt concentration or high temperature, the amount of disorder can be over- or underpredicted depending how these conditions affect the presence of protein disorder.

Among bacterial proteomes, there are a few examples of organisms that seem to utilize a surprisingly large fraction of their disordered amino acids in binding. The three most extreme cases (*Carsonella ruddii*, *Sulcia muelleri* and *Buchnera aphidicola subsp. Cinara cedri*) are marked separately on Figure 8. These are the three smallest complete bacterial proteomes, none of them reaching the size of the smallest archaea proteome. These organisms present extreme cases of streamlined genomes as a result of endosymbiosis [72]–[74]. As these proteomes are very small, the predicted amount of disorder and disordered binding sites are within the false positive range, and should be treated more cautiously.

Eukaryotes tend to appear more consistent both in using larger amount of disordered residues and larger fraction of disordered residues for binding compared to the other two kingdoms (Figure 8). The only notable outlier both in terms of extremely low amount disordered proteins and disordered binding sites is *Encephalitozoon cuniculi*. This organism is the only microsporidian parasite in our dataset and has an extremely small proteome. This lack of complexity and dependence on a eukaryotic host to function might explain the lack of disordered proteins.

**Figure 9:**
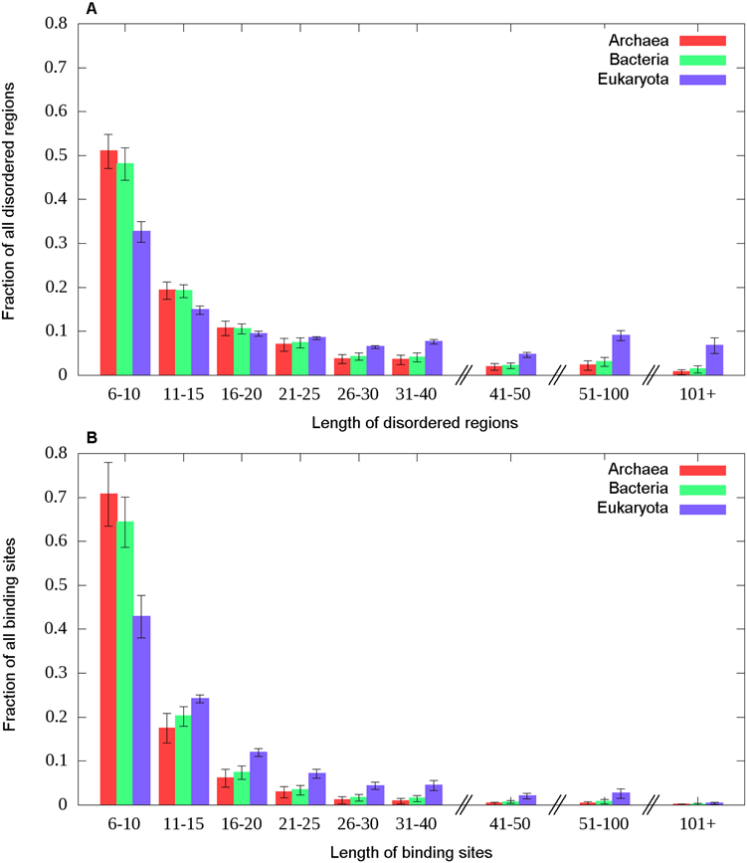
Length distribution of disordered and disordered binding sites in complete proteomes. The length distribution of (A) the disordered protein segments determined by IUPred and (B) predicted disordered binding sites determined by ANCHOR for the 736 complete proteomes available, grouped according to the three kingdoms of life.

The length distributions of the predicted disordered regions and binding sites in the three kingdoms of life was also analyzed and are shown in Figure 9A and 9B, respectively. As complexity increases, longer disordered segments are preferred, and the difference between eukaryota and lower complexity organisms becomes even more apparent for longer regions (over 30 residues). A similar trend can be observed in the length distribution of disordered binding sites. While in archaea and bacteria predicted binding regions are generally below 30 residues, longer binding sites in eukaryota organisms are much more common. There are at least three different effects that can contribute to this phenomenon. First, as the number of binding sites rise there is also an increasing possibility of these binding sites becoming very close to each other or even overlapping with each other. This scenario was demonstrated in the case of the N-terminal domain of p53, as shown in Figure 1. Second, extremely large disordered binding regions may be needed for special functions. Some members of the mucin protein family provide an example for this. Human MUC1 contains a large repeat region (20–120 repeats, one repeat being 20 amino acids long) that enables it to aggregate and to perform its function [75]. As each repeat is correctly identified as a disordered binding site, the whole repeat region is predicted as one large binding region. This mechanism can create binding sites up to the length of several hundreds of residues in extreme cases. Third, we cannot exclude the possibility that longer binding sites are not always segmented by weakly interacting regions like in the case of p27, thus forming long, continuous binding regions. Nevertheless, the majority of predicted binding sites is shorter than 30 residues, although such restriction on the length of disordered binding sites was not enforced.

## Discussion

Regions undergoing disorder-to-order transitions upon binding are essential elements in the molecular recognition process involving disordered proteins. The main property of these binding regions is that they can exist in a disordered state as well as in bound state, adopting at least partially a well-defined conformation. The presence of these two separate states discriminates them from monomeric globular proteins as well as from complexes formed between globular proteins and from disordered proteins in general. They are also expected to differ from dual personality fragments [76], which occur within globular proteins, however, mostly as a result of perturbations of environmental conditions. In this work we aimed to recognize such disordered binding regions from the amino acid sequence. So far, the limited number of well characterized examples hindered the development of general prediction methods. Nevertheless, biophysical considerations suggest that in most cases there is a strong signal in the amino acid sequence highlighting regions involved in coupled folding and binding. These regions are linear in sequence, unlike in the case of globular proteins, where distinct sites in the amino acid sequence are brought together to form the interface for interaction [43]. An additional difference is that binding of disordered proteins is driven by a large enthalpic component to compensate for the entropy penalty due to the loss of conformational freedom [9]. These features result in a relatively short sequence segment containing residues with a pronounced tendency to make interactions, leading to a characteristic sequence signal.

Our approach relies on a basic physical model of disordered binding sites and it is based on modeling the interaction capacity in the free disordered state and in the bound ordered state. Previously, it was shown that ordered proteins can be discriminated from disordered proteins based on estimated pairwise energy content and this approach was implemented in IUPred, a general disorder prediction method [53]. This method takes into account that disorder/order tendency can be modulated by the sequential neighborhood simply at the level of amino acid composition, without attempting to model the specific interactions. Taking it one step further, the same energy estimation calculations were used to identify disordered binding regions in proteins. Our model assumes that the specific properties of disordered binding sites are dictated by the combination of preferences to bind to an ordered protein on the one hand, and the ability to remain in a disordered state in isolation, on the other. Based on this simple model, ANCHOR achieved approximately 67% accuracy at predicting 5% false positive rate (Tables 2–4). Furthermore, this approach was validated by the ability to reproduce the specific amino acid composition of disordered binding sites, that is distinct from that of ordered proteins as well as disordered proteins in general (Table 5).

During binding, the formation of intermolecular contacts is accompanied by the formation or the stabilization of secondary structure elements. The secondary structure composition of the binding sites is highly unequal (Table 6 and Figure 4). The most dominant secondary structure element adopted in the bound conformation is coil, while β strand conformation is rare. Helical conformations are observed as frequently in disordered complexes as in globular proteins [27]. It was found that the adopted secondary structure can be predicted from the amino acid sequence with similar accuracy as in the case of globular proteins, suggesting that the adopted secondary structure can be imprinted into the sequence of the binding motif [27]. The secondary structure observed in the complex can also be dictated by the template structure. An extreme example of this is the C-terminal region of p53 (see Supporting Information), observed in all three secondary structure classes [32]. It is clear that not all of these conformations can be the result of inherent preferences. Interestingly, our prediction method does not seem to be sensitive to the adopted secondary structure conformation and it works with the same accuracy for all secondary structure conformations (Table 6 and Figure 4). This independence of secondary structure elements underlines the generality of ANCHOR. These results also suggest that disordered binding sites can be recognized without taking into account of the adopted secondary structure in the majority of cases. Nevertheless, the details of conformational preferences can be still crucial in selecting the specific binding partner, or determining the kinetic and thermodynamic properties of the associations.

Beside our algorithm, a previously published method called α-MoRF predictor also exploited a general disorder prediction method to recognize short binding elements [48],[52]. Although the direct comparison between the two methods was not possible, because the α-MoRF predictor is not yet publicly available, some basic differences between the two methods should be noted. First, the α-MoRF predictor directly relies on the prediction output of PONDR VXLT, which essentially predicts binding regions as ordered structural elements, and a subsequent neural network is applied to filter out valid disordered binding sites. Although very high accuracies were reported for the performance of the neural network based filtering, the complete method is limited by finding dips based on PONDR VLXT [49]–[51]. Therefore it should be taken into account that this program is a first generation prediction method that was trained on only 15 proteins. In the case of IUPred, dips corresponding to certain binding sites were also observed, although to a smaller extent [48],[53]. This observation, however, is not directly exploited in our prediction method. Instead, the core parameters of the energy prediction of IUPred are used to create three separate scores characterizing three important attributes of disordered binding regions. The second main difference is that ANCHOR is not restricted to a single secondary structure class like the α-MoRF predictor that was trained to recognize only α-helical segments. The example of the C-terminal region of p53 (Figure S2), where four short overlapping regions were shown to bind in different conformations representing all three secondary structure classes, indicates that such restriction can be a serious disadvantage for recognizing some extremely adaptable disordered binding motifs.

An alternative approach for binding site identification is based on the observation that protein-protein interactions are often mediated through short linear motifs (approximately three to eight residues) [77]. Such motifs are defined by a consensus pattern, which captures the key residues involved in function or binding. Prominent examples include the nuclear receptor box motif, MDM2 binding sites, SH2/SH3 domain recognition patterns or 14-3-3 domain binding sites [68]. Although there are known examples of motifs that reside within globular domains, many of them are required to be in a disordered region to function properly and it was suggested that such motifs share many similarities with disordered binding regions [70]. Our preliminary results support previous observations of the partial overlap between short linear motifs and disordered binding segments. Nevertheless, short disordered binding sites and sequence specific linear motifs capture different aspects of certain binding regions. Linear motifs are defined on the basis of a per residue binding strength, and they are specific to a certain partner or to a group of partner molecules. However, such short linear motifs can also occur purely by chance, with no biological significance. Also, sequence patterns alone cannot ensure the accessibility of the site and the potential flexibility of the binding region that could be necessary for the complex formation. Complementary to sequence motifs, ANCHOR aims to capture a broader structural context. Based on their specific structural properties, it can recognize disordered binding regions that are capable of undergoing disorder-to-order transition. The predictions are made without taking into account the partner molecules and are expected to be less sensitive to sequence details. For certain motifs, this molecular environment can be a prerequisite of functionality and could help to identify biologically significant binding motifs.

In our work we assumed, that short binding regions undergoing disorder-to-order transition can be viewed as elementary binding units that are necessary for the molecular recognition. Therefore, such examples were used for the optimization of our method. In accordance with their elementary unit picture, ANCHOR recognized them generally as a single continuous binding site (Figure 5). Regions undergoing disorder-to-order transition, however, are not limited to such short segments as there are several examples of longer disordered segment becoming ordered upon complex formation. Such segments can be as long as 100 residues. However, these longer regions can contain segments which bind only weakly or might not become ordered at all [63],[78],[79]. This segmentation of longer binding regions can occur for structural reasons. The segmentation can prevent the accumulation of the critical amount of residues that would lead to the formation a collapsed structure or non-specific aggregates. The possible functional advantages of the segmented nature of a binding site were demonstrated for the well characterized example of p27. The kinase inhibitory domain of p27 can be divided into several subdomains which dock and fold in a stepwise manner on the surface of the Cdk2-cyclin A complex [19]. These segments can also evolve independently, increasing the repertoire for specificity for different cellular location or species. Intervening segments of higher flexibility are accessible for modifications such as phosphorylations and ubiquitinations. This way p27 can integrate and process various signals to regulate cell proliferation, in which the flexibility and modularity of p27 is essential [63]. The segmented nature of binding is reflected in the prediction output, with predicted binding sites corresponding to the strongly interacting regions (Figure 6 for p27, and Figure S4 for a similar example, calpastatin). In the dataset of longer disordered binding segments, we found this segmentation to be quite general. In these cases, the predicted sites generally give only partial coverage of the PDB structure, and multiple binding sites are predicted in the majority of cases (Figure 5). This suggests that our prediction method is likely to find those sites that interact more strongly, anchoring the disordered segments to their partner protein. While the segmented nature of binding is prominent in the case of long binding regions, to a smaller extent, it can also affect shorter binding regions. Indeed, around 20% of short disordered binding regions are predicted as 2 or 3 segments (Figure 5). This could also account for the significantly lower per residue efficiency compared to the segment based efficiency.

By looking at further individual examples, one can already see remarkable variations in the details of disorder-to-order transitions even within the limited collection that is available today. The adopted conformation in these complexes can be quite different, both in terms of secondary or tertiary structure. Furthermore, the transition to an ordered structure might not be complete [28]. This could leave terminal residues or linker regions flexible and inaccessible to structure determination. It was also suggested that specific binding can be possible even without adopting a well-defined conformation as in the case of the ζ-chain of T-cell receptor [80] (see Figure S6). Differences are also present at the level of the sequence. Some binding regions rely largely on hydrophobic or aromatic residues (MDM2 binding regions, Figure 1), others use proline rich regions (WASp SH3 binding regions, Figure 7). Disordered binding regions can contain conserved linear motifs, while large divergence in sequence was noted in other cases (C terminal domain of histones [81]). These examples represent multiple ways disordered regions can be utilized for binding. A single protein sequence can contain several distinct binding regions, however, a single region can be involved in binding to multiple partners, or use these regions in combination to hub several interactions (p53 – see Figure 1 and Figure S2, WASp – see Figure 7). In an alternative scenario, disorder present in the partner molecules allows to bind a well-folded protein by a large number of proteins (β-catenin [82], Figure S3). Even further variations are expected as the number of examples will grow in the future. Nevertheless, the success of ANCHOR confirms our hypothesis, that despite these differences disordered binding regions have a common property that predispose them for coupled folding and binding.

The occurrence of disordered binding sites is clearly tied to the presence of disordered protein regions. Their relationship was further analyzed at the level of complete proteomes. Previous studies have shown that the amount of predicted disordered regions increases with the complexity of organisms throughout evolution and reaches a high level in multicellular organisms [5],[6]. This increase can be mostly attributed to the appearance of long, domain-sized segments of protein disorder or fully disordered proteins (Figure 9A). Our analysis showed that the amount of disordered binding segments increases in eukaryotes in a similar way, however, their fraction is elevated even compared to disordered regions in general (Figure 8). The observed trend is valid through a wide range of organisms, and occasional exceptions occur either due to adaptation to extreme habitat conditions, or as a result of endosymbiosis. These findings imply that the newly introduced disordered proteins and protein segments mainly serve as a carrier for new binding regions in eukaryotic organisms. The importance of disordered regions in protein-protein interactions is also supported by the increased ratio of disordered proteins among hub proteins [30],[31]. Disordered segments are often involved for complex signaling and regulatory processes [20] such as cell cycle control, gene regulation or signal transduction in the intracellular region of transmembrane proteins [83]. These processes rely on interactions involving multiple partners and high specificity/low affinity interactions, that disordered binding segments can provide by their very nature. The disordered segments can harbor multiple binding sites which can act relatively independently. In other cases segmented binding sites can be involved in simultaneous binding to larger complexes. Overlapping binding sites (such in the case of p53 N and C terminal regions) suggest competition between binding partners. We are only beginning to comprehend how disordered binding regions are exploited to provide versatile interaction sites in proteins.

In conclusion, disordered binding regions represent a specific subclass of disordered proteins that can undergo a disorder-to-order transition upon binding. These binding sites generally have distinct properties both structurally and functionally. Due to the inherent flexibility, these regions are difficult to study experimentally [84], making specific prediction methods even more valuable. While there are several methods available for prediction of disordered regions [85],[86], recognizing disordered binding sites was regarded as a more challenging problem [9] due to the limited number of well-characterized examples. In this work we report a general method to recognize disordered binding sites based on a basic biophysical model. Our method relies on a simple energy estimation procedure that was developed earlier for the IUPred disorder prediction method. This way, the problem of small datasets can be largely avoided. We showed that these regions can be characterized by highly disordered sequential neighborhood, unfavorable intrachain energies and more favorable interaction energies with a globular partner. The combination of these properties allowed the recognition of disordered binding sites independent of their secondary structure or amino acid composition, underlining the generality of the method. As such binding sites are essential functional elements of disordered proteins, their prediction directly provides information about functionally important residues in these proteins. In this way, ANCHOR broadens the repertoire of prediction methods for functional sites in proteins aiming to decrease the large number of unannotated sequences [87]. Generally, the complete understanding of protein-protein interactions involving disordered binding sites requires the knowledge of their partners as well as possible post-translational modifications that can influence their binding. While predictions can be made even without taking the partner molecule into account, certain cases might require incorporating the specific feature of the partner. Nevertheless, our method can provide the starting point for such scientific explorations, by finding potential regions involved in such binding.

## Methods

### Databases

The primary source of data for the present analysis is a carefully assembled dataset of binding regions undergoing disorder-to-order transition. The strict requirement of the experimental verification of both the disordered status in isolation and the formation of an ordered structure in complex distinguishes our dataset from a previously collected dataset for disordered binding regions [88]. The length of disordered regions involved in the binding can vary on a large scale. In the case of longer regions it is not guaranteed that each residue is equally important for binding, therefore complexes of short disordered regions were treated separately, and only these were used for tuning the method.

#### Short disordered complexes

Complexes from the PDB [89] were collected by scanning the chains in the PDB entries against the Disprot database [90]. A complex was accepted if it consisted of a chain with length between 10 and 30 residues that was found in the Disprot database as part of an annotated disordered segment and at least one interacting partner that was at least 40 residues long. Furthermore, complexes containing transmembrane proteins, RNA or DNA, chimeras, disulfide bonds between the disordered and ordered chains or a large number of unknown residues (marked with an X) were excluded. A few experimentally verified disordered complexes missing from Disprot were added to this set [42], [43], [62], [91]–[93]. A sequence similarity filter of 50% has also been applied to remove closely related proteins or protein segments. This procedure yielded a set of 46 complexes that are listed in Dataset S1.

#### Long disordered complexes

Complexes containing long disordered chains were collected in the same fashion as short ones but with different criteria for the length of the interacting partners. Here the length of the disordered chains was required to be at least 30 residues and they had to have an interacting partner of 70 residues or more. The resulting set of 28 complexes is listed in Dataset S2.

#### α-MoRFs dataset

This dataset originally consisted of 53 complexes [48]. Complexes that were contained in our Short disordered complexes dataset as well were excluded in order to get a truly independent set. Three complexes were further removed from the remainder since one of them is part of the ribosome subunit S23 and the other two can be found in the PBD with structures containing only the disordered chain – that is they are presumably capable of folding on their own. The rationale behind this exclusion is that our predictor is neither trained to recognize RNA/DNA-protein interactions nor to identify globular-globular interfaces. This left 40 complexes in total.

#### Globular proteins

Globular proteins were collected from PDB entries that had only one chain of at least 30 residues [53]. Also transmembrane proteins and complexes with RNA/DNA were filtered out. This dataset contains 553 proteins and is presented in Dataset S3.

#### Ordered complexes

This set contains protein complexes that consist of two partners both of which are ordered. These data were taken from the literature [43]. The dataset does not include cases of crystal packing dimers, chimeras and fragments and consists of 72 complexes (Dataset S4).

#### Disordered proteins

For the analysis of disordered proteins and protein segments the 3.7 version of Disprot database was used (http://www.disprot.org/) [90], considering only annotated disordered segments of 10 residues or longer.

### Parameter optimization

The optimal parameters were determined by a three fold cross-validation, by dividing both our negative and positive datasets (Globular proteins and Short disordered complexes, respectively) into three parts. In each turn we used two parts for training and the remaining part for testing. To avoid any bias, the different subsets were chosen such that the distribution of chain lengths in both the positive and negative sets and the distribution of secondary structure types in the positive set were approximately the same. Our approach relies on IUPred, a general disorder prediction method, and its energy predictor matrix. These parameters (ie. the elements of the energy predictor matrix) have been determined earlier, independently of disordered binding regions. Only five additional parameters, *w_1_*, *w_2_*, *p_1_*, *p_2_* and *p_3_* were optimized for this specific problem and were selected by a grid search procedure. Specifically, *w_1_* was varied in the range of 20 to 100 in steps of 10 (giving 9 possible values), *w_2_* was varied in the range of 5 to 35 in steps of 2 (giving 16 possible values), and *p_1_*, *p_2_* and *p_3_* was selected from 1000 sets of randomly generated values. Taking into account that the prediction performance is insensitive to the norm and the sign of the vector corresponding to the *p_1_*, *p_2_* and *p_3_* values, the search was restricted to 1000 random sets that were evenly distributed on the surface of the upper half of the unit sphere. This means that *p_1_* and *p_2_* were randomly selected from the interval [−1;1] and *p_3_* was selected from the interval [0;1] in a way that the sum of their squares is always equal to 1. This yielded 1000 different (*p_1_*, *p_2_*, *p_3_*) combinations. These, combined with all possible values of *w_1_* and *w_2_* gave 144,000 different parameter sets in total. These were considered in order to select the optimal one, containing the five optimal parameters for each round of the cross-validation.

To quantify the performance of the predictor given a set of parameters we calculated the True Positive Rate (TPR) at False Positive Rates (FPR) fixed at 5% calculated on globular proteins as the negative set. However, a full characterization of the performance of the algorithm would also require a set of disordered proteins that are known *not* to bind to globular proteins. Unfortunately, such dataset cannot be constructed since there is hardly any way to give evidence for a protein that it does not contain binding sites. This problem was addressed by calculating the fraction of amino acids that are predicted as binding sites in general disordered regions of Disprot database that are correctly recognized as disordered by IUPred. This fraction was denoted as *F*. Optimal parameters should combine high TPR with low *F* at the expense of very low FPR.

During optimization of the algorithm, the performance on three different datasets needed to be monitored at the same time (set of globular proteins, set of disordered binding sites and Disprot). The best parameter set was chosen manually, by reducing the parameter set in a step-wise manner based on the following steps:

1, Calculate TPR (at fixed FPR = 5%) and F for each of the 144,000 candidate sets of parameters

2, Discard all for which F>50%

3, Discard all for which TPR<60%

4, From the remainder choose the 20 for which the difference between TPR and F is the largest

5, Choose the one for which TPR is maximal (the TPR-F difference among these 20 sets vary only within a range of less then 0.02 so that is not a good measure to choose the best one)

The negative and positive sets were divided into three parts, resulting in three different optimal parameter sets. The final predictor algorithm is constructed by averaging these three outputs. As the training sets only contained binding regions of at least 10 amino acids and we aim to identify at least 5 residues of each region, all predicted binding sites were removed that did not exceed 5 consecutive residues. A schematic figure of the training procedure is given in Figure S1.

### Availability

ANCHOR is available upon request from the authors.

## Supporting Information

Dataset S1 46 complexes of short disordered and long globular proteins. Column 4 contains the secondary structure type of the bound disordered chains based on the structure found in the PDB record as defined in Data and Methods. Thick lines separate the three groups used during parameter optimization.
(0.07 MB DOC)

Dataset S2 28 complexes of long disordered and long globular proteins. Column 4 contains the secondary structure type of the bound disordered chains based on the structure found in the PDB record as defined in Data and Methods.
(0.05 MB DOC)

Dataset S3 553 monomeric globular proteins that were used as a negative dataset [2]. Columns correspond to the grouping used during parameter optimization.
(0.20 MB DOC)

Dataset S4 72 complexes of ordered proteins [3]. The interaction is considered between the shortest chains and its interaction partners.
(0.08 MB DOC)

Dataset S5 The 53 complete archaea proteomes available from SwissProt (ftp://ftp.expasy.org/) used for full proteome scans. The fraction of total amino acids in disordered regions and the fraction of disordered amino acids in disordered binding sites are indicated together for each organism.
(0.09 MB DOC)

Dataset S6 The 639 complete bacteria proteomes available from SwissProt (ftp://ftp.expasy.org/) used for full proteome scans. The fraction of total amino acids in disordered regions and the fraction of disordered amino acids in disordered binding sites are indicated together for each organism.
(0.86 MB DOC)

Dataset S7 The 44 complete eukaryota proteomes available from SwissProt (ftp://ftp.expasy.org/) used for full proteome scans. The fraction of total amino acids in disordered regions and the fraction of disordered amino acids in disordered binding sites are indicated together for each organism.
(0.08 MB DOC)

Figure S1 Development of ANCHOR. In the first step, our Short Disordered Binding Sites dataset and Globular Proteins dataset (positive and negative datasets) are split up and only 2/3 is used in the subsequential steps. Then a parameter set (w1, w2, p1, p2, p3) is selected from the 144,000 random ones. This parameter set is used to calculate S, Eint and Egain for every position in every sequence in the three input datasets using the fixed energy predictor matrix P (see Theory). Based on this calculations the evaluating measures are calculated: TPR is calculated on Short Disordered Binding Sites, FPR is calculated on Globular Proteins and F is calculated on Disordered Proteins. Based on these measures, the best parameter set out of 144,000 is chosen (see Data and Methods). Then this parameter set is evaluated on the remaining one third of the datasets. These results are reported in Table 3. This procedure is repeated for all three subsets of Short Disordered Binding Sites and Globular Proteins. The output of the three optimized predictors are combined into one final predictor by averaging their output.
(0.05 MB PPT)

Figure S2 ANCHOR prediction output for the C-terminal domain of human p53. Prediction for the C-terminal disordered domain of human p53. The regulatory binding site around residues 375–390 is able to adopt all three secondary structural elements upon binding to globular partners [4].
(0.04 MB TIF)

Figure S3 ANCHOR prediction output for Tcf4. Prediction output for transcription factor Tcf4 (blue) together with the number of atomic contacts (green) determined in the complexed form with Beta-catenin (PDB ID: 2gl7 [5]). Beta-catenin is known to bind several disordered binding regions.
(0.03 MB TIF)

Figure S4 ANCHOR prediction output for human calpastatin. Prediction output for the I. domain of human calpastatin. Subdomains A. B and C (grey boxes) are known to bind to calpain and inhibit it. Subdomains A and C bind via a preformed alpha-helix. while subdomain B does not exhibit strong structural preference in solution [6].
(0.04 MB TIF)

Figure S5 ANCHOR prediction output for the KID domain of CREB. Prediction output for the KID domain of CREB. The region marked with a grey box interacts with the KIX domain of CBP via two preformed alpha-helices [7].
(0.03 MB TIF)

Figure S6 ANCHOR prediction output for the ζ-chain of T-cell receptor. Prediction output for the zeta-chain of the T-cell receptor. The transmembrane region is marked with red box and the three intracellular ITAM regions are marked with blue boxes.
(0.12 MB TIF)

Protocol S1 Protocol including references for the Supporting Information.
(0.04 MB DOC)

## References

[1] Wright PE, Dyson HJ (1999). Intrinsically unstructured proteins: re-assessing the protein
structure-function paradigm.. J Mol Biol.

[2] Dyson HJ, Wright PE (2005). Intrinsically unstructured proteins and their functions.. Nat Rev Mol Cell Biol.

[3] Dunker AK, Lawson JD, Brown CJ, Williams RM, Romero P (2001). Intrinsically disordered protein.. J Mol Graph Model.

[4] Tompa P (2002). Intrinsically unstructured proteins.. Trends Biochem Sci.

[5] Dunker AK, Obradovic Z, Romero P, Garner EC, Brown CJ (2000). Intrinsic protein disorder in complete genomes.. Genome Inform Ser Workshop Genome Inform.

[6] Ward JJ, Sodhi JS, McGuffin LJ, Buxton BF, Jones DT (2004). Prediction and functional analysis of native disorder in proteins
from the three kingdoms of life.. J Mol Biol.

[7] Xie H, Vucetic S, Iakoucheva LM, Oldfield CJ, Dunker AK (2007). Functional anthology of intrinsic disorder. 1. Biological
processes and functions of proteins with long disordered regions.. J Proteome Res.

[8] Tompa P (2005). The interplay between structure and function in intrinsically
unstructured proteins.. FEBS Lett.

[9] Galea CA, Wang Y, Sivakolundu SG, Kriwacki RW (2008). Regulation of cell division by intrinsically unstructured
proteins: intrinsic flexibility, modularity, and signaling conduits.. Biochemistry.

[10] Chen J, Kanai Y, Cowan NJ, Hirokawa N (1992). Projection domains of MAP2 and tau determine spacings between
microtubules in dendrites and axons.. Nature.

[11] Linke WA, Kulke M, Li H, Fujita-Becker S, Neagoe C (2002). PEVK domain of titin: an entropic spring with actin-binding
properties.. J Struct Biol.

[12] Mukhopadhyay R, Kumar S, Hoh JH (2004). Molecular mechanisms for organizing the neuronal cytoskeleton.. Bioessays.

[13] Hoh JH (1998). Functional protein domains from the thermally driven motion of
polypeptide chains: a proposal.. Proteins.

[14] Alber F, Dokudovskaya S, Veenhoff LM, Zhang W, Kipper J (2007). The molecular architecture of the nuclear pore complex.. Nature.

[15] Bruschweiler R, Liao X, Wright PE (1995). Long-range motional restrictions in a multidomain zinc-finger
protein from anisotropic tumbling.. Science.

[16] Dyson HJ, Wright PE (2002). Coupling of folding and binding for unstructured proteins.. Curr Opin Struct Biol.

[17] Uversky VN (2002). Natively unfolded proteins: a point where biology waits for
physics.. Protein Sci.

[18] Demarest SJ, Martinez-Yamout M, Chung J, Chen H, Xu W (2002). Mutual synergistic folding in recruitment of CBP/p300 by p160
nuclear receptor coactivators.. Nature.

[19] Lacy ER, Filippov I, Lewis WS, Otieno S, Xiao L (2004). p27 binds cyclin-CDK complexes through a sequential mechanism
involving binding-induced protein folding.. Nat Struct Mol Biol.

[20] Uversky VN, Oldfield CJ, Dunker AK (2005). Showing your ID: intrinsic disorder as an ID for recognition,
regulation and cell signaling.. J Mol Recognit.

[21] Liu J, Perumal NB, Oldfield CJ, Su EW, Uversky VN (2006). Intrinsic disorder in transcription factors.. Biochemistry.

[22] Iakoucheva LM, Brown CJ, Lawson JD, Obradovic Z, Dunker AK (2002). Intrinsic disorder in cell-signaling and cancer-associated
proteins.. J Mol Biol.

[23] Fuxreiter M, Tompa P, Simon I, Uversky VN, Hansen JC (2008). Malleable machines take shape in eukaryotic transcriptional
regulation.. Nat Chem Biol.

[24] Dunker AK, Garner E, Guilliot S, Romero P, Albrecht K (1998). Protein disorder and the evolution of molecular recognition:
theory, predictions and observations.. Pac Symp Biocomput.

[25] Kriwacki RW, Hengst L, Tennant L, Reed SI, Wright PE (1996). Structural studies of p21Waf1/Cip1/Sdi1 in the free and
Cdk2-bound state: conformational disorder mediates binding diversity.. Proc Natl Acad Sci U S A.

[26] Bienkiewicz EA, Adkins JN, Lumb KJ (2002). Functional consequences of preorganized helical structure in the
intrinsically disordered cell-cycle inhibitor p27(Kip1).. Biochemistry.

[27] Fuxreiter M, Simon I, Friedrich P, Tompa P (2004). Preformed structural elements feature in partner recognition by
intrinsically unstructured proteins.. J Mol Biol.

[28] Tompa P, Fuxreiter M (2008). Fuzzy complexes: polymorphism and structural disorder in
protein-protein interactions.. Trends Biochem Sci.

[29] Spolar RS, Record MT (1994). Coupling of local folding to site-specific binding of proteins to
DNA.. Science.

[30] Dosztanyi Z, Chen J, Dunker AK, Simon I, Tompa P (2006). Disorder and sequence repeats in hub proteins and their
implications for network evolution.. J Proteome Res.

[31] Haynes C, Oldfield CJ, Ji F, Klitgord N, Cusick ME (2006). Intrinsic disorder is a common feature of hub proteins from four
eukaryotic interactomes.. PLoS Comput Biol.

[32] Oldfield CJ, Meng J, Yang JY, Yang MQ, Uversky VN (2008). Flexible nets: disorder and induced fit in the associations of
p53 and 14-3-3 with their partners.. BMC Genomics.

[33] Iakoucheva LM, Radivojac P, Brown CJ, O'Connor TR, Sikes JG (2004). The importance of intrinsic disorder for protein phosphorylation.. Nucleic Acids Res.

[34] Albert R, Jeong H, Barabási AL (2000). Error and attack tolerance of complex networks.. Nature.

[35] Uversky VN, Oldfield CJ, Dunker AK (2008). Intrinsically disordered proteins in human diseases: introducing
the D2 concept.. Annu Rev Biophys.

[36] Vogelstein B, Lane D, Levine AJ (2000). Surfing the p53 network.. Nature.

[37] Cheng Y, LeGall T, Oldfield CJ, Dunker AK, Uversky VN (2006). Abundance of intrinsic disorder in protein associated with
cardiovascular disease.. Biochemistry.

[38] Frankfort SV, Tulner LR, van Campen JP, Verbeek MM, Jansen RW (2008). Amyloid beta protein and tau in cerebrospinal fluid and plasma as
biomarkers for dementia: a review of recent literature.. Curr Clin Pharmacol.

[39] Waxman EA, Giasson BI (2008). Molecular mechanisms of alpha-synuclein neurodegeneration.. Biochim Biophys Acta.

[40] Marc D, Mercey R, Lantier F (2007). Scavenger, transducer, RNA chaperone? What ligands of the prion
protein teach us about its function.. Cell Mol Life Sci.

[41] Cheng Y, LeGall T, Oldfield CJ, Mueller JP, Van YY (2006). Rational drug design via intrinsically disordered protein.. Trends Biotechnol.

[42] Gunasekaran K, Tsai CJ, Nussinov R (2004). Analysis of ordered and disordered protein complexes reveals
structural features discriminating between stable and unstable monomers.. J Mol Biol.

[43] Meszaros B, Tompa P, Simon I, Dosztanyi Z (2007). Molecular principles of the interactions of disordered proteins.. J Mol Biol.

[44] Vacic V, Oldfield CJ, Mohan A, Radivojac P, Cortese MS (2007). Characterization of molecular recognition features, MoRFs, and
their binding partners.. J Proteome Res.

[45] Vucetic S, Brown CJ, Dunker AK, Obradovic Z (2003). Flavors of protein disorder.. Proteins.

[46] Schlessinger A, Punta M, Rost B (2007). Natively unstructured regions in proteins identified from contact
predictions.. Bioinformatics.

[47] Garner E, Romero P, Dunker AK, Brown C, Obradovic Z (1999). Predicting binding regions within disordered proteins.. Genome Inform Ser Workshop Genome Inform.

[48] Cheng Y, Oldfield CJ, Meng J, Romero P, Uversky VN (2007). Mining alpha-helix-forming molecular recognition features with
cross species sequence alignments.. Biochemistry.

[49] Romero, Obradovic, Dunker K (1997). Sequence data analysis for long disordered regions prediction in
the calcineurin family.. Genome Inform Ser Workshop Genome Inform.

[50] Romero P, Obradovic Z, Li X, Garner EC, Brown CJ (2001). Sequence complexity of disordered protein.. Proteins.

[51] Li X, Romero P, Rani M, Dunker AK, Obradovic Z (1999). Predicting protein disorder for N-, C-, and internal regions.. Genome Inform Ser Workshop Genome Inform.

[52] Oldfield CJ, Cheng Y, Cortese MS, Romero P, Uversky VN (2005). Coupled folding and binding with alpha-helix-forming molecular
recognition elements.. Biochemistry.

[53] Dosztanyi Z, Csizmok V, Tompa P, Simon I (2005). The pairwise energy content estimated from amino acid composition
discriminates between folded and intrinsically unstructured proteins.. J Mol Biol.

[54] Dosztanyi Z, Csizmok V, Tompa P, Simon I (2005). IUPred: web server for the prediction of intrinsically
unstructured regions of proteins based on estimated energy content.. Bioinformatics.

[55] Chumakov PM (2007). Versatile functions of p53 protein in multicellular organisms.. Biochemistry (Mosc).

[56] Dawson R, Muller L, Dehner A, Klein C, Kessler H (2003). The N-terminal domain of p53 is natively unfolded.. J Mol Biol.

[57] Kussie PH, Gorina S, Marechal V, Elenbaas B, Moreau J (1996). Structure of the MDM2 oncoprotein bound to the p53 tumor
suppressor transactivation domain.. Science.

[58] Bochkareva E, Kaustov L, Ayed A, Yi GS, Lu Y (2005). Single-stranded DNA mimicry in the p53 transactivation domain
interaction with replication protein A.. Proc Natl Acad Sci U S A.

[59] Di Lello P, Jenkins LM, Jones TN, Nguyen BD, Hara T (2006). Structure of the Tfb1/p53 complex: Insights into the interaction
between the p62/Tfb1 subunit of TFIIH and the activation domain of p53.. Mol Cell.

[60] Fawcett T (2006). An introduction to ROC analysis.. Pattern Recognit Lett.

[61] Kabsch W, Sander C (1983). Dictionary of protein secondary structure: pattern recognition of
hydrogen-bonded and geometrical features.. Biopolymers.

[62] Russo AA, Jeffrey PD, Patten AK, Massague J, Pavletich NP (1996). Crystal structure of the p27Kip1 cyclin-dependent-kinase
inhibitor bound to the cyclin A-Cdk2 complex.. Nature.

[63] Galea CA, Nourse A, Wang Y, Sivakolundu SG, Heller WT (2008). Role of intrinsic flexibility in signal transduction mediated by
the cell cycle regulator, p27 Kip1.. J Mol Biol.

[64] Ochs HD, Notarangelo LD (2005). Structure and function of the Wiskott-Aldrich syndrome protein.. Curr Opin Hematol.

[65] Kim AS, Kakalis LT, Abdul-Manan N, Liu GA, Rosen MK (2000). Autoinhibition and activation mechanisms of the Wiskott-Aldrich
syndrome protein.. Nature.

[66] Marchand JB, Kaiser DA, Pollard TD, Higgs HN (2001). Interaction of WASP/Scar proteins with actin and vertebrate
Arp2/3 complex.. Nat Cell Biol.

[67] Abdul-Manan N, Aghazadeh B, Liu GA, Majumdar A, Ouerfelli O (1999). Structure of Cdc42 in complex with the GTPase-binding domain of
the ‘Wiskott-Aldrich syndrome’ protein.. Nature.

[68] Puntervoll P, Linding R, Gemund C, Chabanis-Davidson S, Mattingsdal M (2003). ELM server: A new resource for investigating short functional
sites in modular eukaryotic proteins.. Nucleic Acids Res.

[69] Tong AH, Drees B, Nardelli G, Bader GD, Brannetti B (2002). A combined experimental and computational strategy to define
protein interaction networks for peptide recognition modules.. Science.

[70] Fuxreiter M, Tompa P, Simon I (2007). Local structural disorder imparts plasticity on linear motifs.. Bioinformatics.

[71] Preston CM, Wu KY, Molinski TF, DeLong EF (1996). A psychrophilic crenarchaeon inhabits a marine sponge:
Cenarchaeum symbiosum gen. nov., sp. nov.. Proc Natl Acad Sci U S A.

[72] Perez-Brocal V, Gil R, Ramos S, Lamelas A, Postigo M (2006). A small microbial genome: the end of a long symbiotic
relationship?. Science.

[73] Nakabachi A, Yamashita A, Toh H, Ishikawa H, Dunbar HE (2006). The 160-kilobase genome of the bacterial endosymbiont Carsonella.. Science.

[74] Wu D, Daugherty SC, Van Aken SE, Pai GH, Watkins KL (2006). Metabolic complementarity and genomics of the dual bacterial
symbiosis of sharpshooters.. PLoS Biol.

[75] Hanisch FG, Muller S (2000). MUC1: the polymorphic appearance of a human mucin.. Glycobiology.

[76] Zhang Y, Stec B, Godzik A (2007). Between order and disorder in protein structures: analysis of
“dual personality” fragments in proteins.. Structure.

[77] Neduva V, Russell RB (2005). Linear motifs: evolutionary interaction switches.. FEBS Lett.

[78] Kiss R, Kovács D, Tompa P, Perczel A (2008). Local structural preferences of calpastatin, the intrinsically
unstructured protein inhibitor of calpain.. Biochemistry.

[79] Hurley TD, Yang J, Zhang L, Goodwin KD, Zou Q (2007). Structural basis for regulation of protein phosphatase 1 by
inhibitor-2.. J Biol Chem.

[80] Sigalov A, Aivazian D, Stern L (2004). Homooligomerization of the cytoplasmic domain of the T cell
receptor zeta chain and of other proteins containing the immunoreceptor
tyrosine-based activation motif.. Biochemistry.

[81] Hansen JC, Lu X, Ross ED, Woody RW (2006). Intrinsic protein disorder, amino acid composition, and histone
terminal domains.. J Biol Chem.

[82] Sampietro J, Dahlberg CL, Cho US, Hinds TR, Kimelman D (2006). Crystal structure of a beta-catenin/BCL9/Tcf4 complex.. Mol Cell.

[83] De Biasio A, Guarnaccia C, Popovic M, Uversky VN, Pintar A (2008). Prevalence of intrinsic disorder in the intracellular region of
human single-pass type I proteins: the case of the notch ligand delta-4.. J Proteome Res.

[84] Dosztanyi Z, Sandor M, Tompa P, Simon I (2007). Prediction of protein disorder at the domain level.. Curr Protein Pept Sci.

[85] Dosztanyi Z, Tompa P (2008). Prediction of protein disorder.. Methods Mol Biol.

[86] Ferron F, Longhi S, Canard B, Karlin D (2006). A practical overview of protein disorder prediction methods.. Proteins.

[87] Rost B, Liu J, Nair R, Wrzeszczynski KO, Ofran Y (2003). Automatic prediction of protein function.. Cell Mol Life Sci.

[88] Mohan A, Oldfield CJ, Radivojac P, Vacic V, Cortese MS (2006). Analysis of molecular recognition features (MoRFs).. J Mol Biol.

[89] Berman HM, Westbrook J, Feng Z, Gilliland G, Bhat TN (2000). The Protein Data Bank.. Nucleic Acids Res.

[90] Sickmeier M, Hamilton JA, LeGall T, Vacic V, Cortese MS (2007). DisProt: the Database of Disordered Proteins.. Nucleic Acids Res.

[91] Huber AH, Weis WI (2001). The structure of the beta-catenin/E-cadherin complex and the
molecular basis of diverse ligand recognition by beta-catenin.. Cell.

[92] Hertzog M, van Heijenoort C, Didry D, Gaudier M, Coutant J (2004). The beta-thymosin/WH2 domain; structural basis for the switch
from inhibition to promotion of actin assembly.. Cell.

[93] Sorenson MK, Ray SS, Darst SA (2004). Crystal structure of the flagellar sigma/anti-sigma complex
sigma(28)/FlgM reveals an intact sigma factor in an inactive conformation.. Mol Cell.

[94] Fauchere JL, Charton M, Kier LB, Verloop A, Pliska V (1988). Amino acid side chain parameters for correlation studies in
biology and pharmacology.. Int J Pept Protein Res.

