# Supplementary material for: Prediction of Protein Binding Regions in Disordered Proteins
Source: PLoS Comput Biol. 2009 May 1;5(5):e1000376. doi: 10.1371/journal.pcbi.1000376 (PMC2671142; doi:10.1371/journal.pcbi.1000376)
Supplement: Dataset S1 — 46 complexes of short disordered and long globular proteins. Column 4 contains the secondary structure type of the bound disordered chains based on the structure found in the PDB record as defined in Data and Methods. Thick lines separate the three groups used during parameter optimization. (0.07 MB DOC) [file pcbi.1000376.s001.doc]

| PDB ID | Disordered chain ID | Chain ID of interacting partner(s) | Secondary structure type |
| --- | --- | --- | --- |
| 1a3b | I | H | C |
| 1axc | B | A | E |
| 1ee5 | B | A | C |
| 1ej4 | B | A | H |
| 1h2k | S | A | H+C |
| 1hv2 | B | A | H |
| 1j2x | B | A | H |
| 1k2d | P | AB | C |
| 1mv0 | A | B | C |
| 1ozs | B | A | C |
| 1p16 | D | AB | C |
| 1sb0 | B | A | H |
| 1t08 | C | A | C |
| 2gl7 | E | D | H+C |
| 2phe | C | AB | H |
| 3b71 | D | A | H |
| 1dpj | B | A | H |
| 1fv1 | C | AB | C |
| 1i8h | A | B | C |
| 1iwq | B | A | C |
| 1mxl | I | C | H |
| 1o9a | B | A | C |
| 1p22 | C | A | C |
| 1r1r | D | A | C |
| 1sqk | B | A | H+C |
| 1tce | B | A | C |
| 1ycq | B | A | H |
| 2b3g | B | A | H |
| 2fym | B | AC | C |
| 2iv8 | P | A | H |
| 2nl9 | B | A | H |
| 1a81 | B | A | C |
| 1apm | I | E | H+C |
| 1cqt | I | A | H+C |
| 1dt7 | X | A | H |
| 1emu | B | A | H |
| 1h8b | B | A | H |
| 1kdx | B | A | H |
| 1lm8 | H | V | C |
| 1nx1 | C | A | H |
| 1p4b | P | LH | H |
| 1un0 | C | A | C |
| 2gsi | X | CD | C |
| 2ivz | E | A | C |
| 2oq1 | B | A | C |
| 2pg1 | I | ABFG | E |
