# Supplementary material for: Prediction of Protein Binding Regions in Disordered Proteins
Source: PLoS Comput Biol. 2009 May 1;5(5):e1000376. doi: 10.1371/journal.pcbi.1000376 (PMC2671142; doi:10.1371/journal.pcbi.1000376)
Supplement: Dataset S2 — 28 complexes of long disordered and long globular proteins. Column 4 contains the secondary structure type of the bound disordered chains based on the structure found in the PDB record as defined in Data and Methods. (0.05 MB DOC) [file pcbi.1000376.s002.doc]

| PDB ID | Disordered chain | Interacting partner(s) | Secondary Structure type |
| --- | --- | --- | --- |
| 1cee | B | A | H+E+C |
| 1dev | B | A | C |
| 1dow | B | A | H |
| 1fqj | C | AB | H |
| 1g3j | B | A | H+C |
| 1hrt | I | H | C |
| 1i7w | B | A | H+C |
| 1j2j | B | A | H |
| 1jsu | C | AB | H+E+C |
| 1kil | C | ABE | H |
| 1kil | D | ABE | H |
| 1l8c | B | A | H+C |
| 1p4q | A | B | H+C |
| 1pq1 | B | A | H |
| 1q68 | A | B | H |
| 1rf8 | B | A | H+C |
| 1sc5 | B | A | H |
| 1sqq | I | A | C |
| 1tba | A | B | C |
| 1th1 | C | A | H+C |
| 1xtg | B | A | H+C |
| 1ymh | E | A | H+E |
| 1zoq | C | AB | H |
| 2a6q | B | ACEF | H+E |
| 2auh | B | A | H+C |
| 2c1t | D | B | C |
| 2cly | C | ABE | H+C |
| 2o8a | I | A | H+C |
