# Supplementary material for: Prediction of Protein Binding Regions in Disordered Proteins
Source: PLoS Comput Biol. 2009 May 1;5(5):e1000376. doi: 10.1371/journal.pcbi.1000376 (PMC2671142; doi:10.1371/journal.pcbi.1000376)
Supplement: Dataset S3 — 553 monomeric globular proteins that were used as a negative dataset [2]. Columns correspond to the grouping used during parameter optimization. (0.20 MB DOC) [file pcbi.1000376.s003.doc]

| PDB IDs of proteins in group 1 | PDB IDs of proteins in group 2 | PDB IDs of proteins in group 3 |
| --- | --- | --- |
| 1a7m | 16pk | 1a0i |
| 1aat | 1a3c | 1a1x |
| 1ab3 | 1a3k | 1a1z |
| 1abv | 1a9v | 1a41 |
| 1agg | 1ah9 | 1a62 |
| 1al3 | 1ako | 1a8d |
| 1amf | 1am2 | 1aa3 |
| 1amx | 1amm | 1aba |
| 1aod | 1aoy | 1ads |
| 1at0 | 1apj | 1afp |
| 1atx | 1b0u | 1ah5 |
| 1ax3 | 1b6f | 1aho |
| 1ax8 | 1ba5 | 1alu |
| 1ayj | 1bbs | 1auq |
| 1b6g | 1bc4 | 1axh |
| 1bgk | 1bdo | 1b0x |
| 1bhu | 1bea | 1b22 |
| 1bj8 | 1beg | 1b87 |
| 1bqc | 1bf8 | 1bak |
| 1br9 | 1bkb | 1bax |
| 1brt | 1bkr | 1bfe |
| 1btn | 1bnb | 1bgf |
| 1bvh | 1bo9 | 1bgw |
| 1bw6 | 1boe | 1br0 |
| 1by1 | 1bsn | 1buo |
| 1by2 | 1bu2 | 1buy |
| 1byw | 1bu9 | 1bys |
| 1c01 | 1bx7 | 1c05 |
| 1c1k | 1byi | 1c1l |
| 1c20 | 1c3m | 1cb1 |
| 1c25 | 1c4e | 1ccz |
| 1c2a | 1c8e | 1ce4 |
| 1c44 | 1ccv | 1cew |
| 1c6w | 1cdb | 1cl3 |
| 1c8z | 1cen | 1co1 |
| 1c9f | 1cex | 1cxq |
| 1cby | 1cj5 | 1d1n |
| 1cc8 | 1ckv | 1d6b |
| 1cdq | 1cn7 | 1d6t |
| 1cfe | 1coo | 1d8b |
| 1chd | 1cqy | 1ddw |
| 1clh | 1d2p | 1dec |
| 1cmr | 1d5t | 1df3 |
| 1cne | 1d9n | 1dg3 |
| 1cnv | 1dcz | 1dk8 |
| 1cok | 1dgn | 1dkc |
| 1cou | 1di6 | 1dqc |
| 1cv8 | 1dk3 | 1dqg |
| 1cy4 | 1dl0 | 1dto |
| 1cyw | 1dnl | 1dtp |
| 1d1h | 1dzo | 1dv5 |
| 1d8j | 1e4s | 1dxy |
| 1dcs | 1e5u | 1e0l |
| 1df6 | 1e6b | 1e41 |
| 1dgu | 1ea2 | 1e5c |
| 1dhn | 1ed1 | 1e8p |
| 1dv0 | 1ed7 | 1eik |
| 1dzc | 1ehs | 1eit |
| 1e3t | 1eij | 1ejg |
| 1e68 | 1em2 | 1enw |
| 1e6u | 1emx | 1eok |
| 1edg | 1en2 | 1ep0 |
| 1eg2 | 1eoq | 1es6 |
| 1egu | 1erd | 1es9 |
| 1egx | 1es5 | 1euw |
| 1ei0 | 1eu8 | 1evz |
| 1eo0 | 1ew4 | 1ews |
| 1eo1 | 1eyh | 1f0i |
| 1ery | 1f1e | 1f6v |
| 1evs | 1f32 | 1faz |
| 1ewi | 1f53 | 1fgy |
| 1f0z | 1f7c | 1fit |
| 1f40 | 1fbr | 1fsz |
| 1f4p | 1fg5 | 1fy9 |
| 1f94 | 1fjj | 1fyc |
| 1fa2 | 1fvg | 1fyx |
| 1fct | 1fvl | 1g4f |
| 1fen | 1fw9 | 1g5z |
| 1fex | 1fwo | 1g6x |
| 1fg7 | 1fzt | 1g7o |
| 1fkm | 1g11 | 1g84 |
| 1fl0 | 1g1z | 1g9p |
| 1fo8 | 1g2b | 1gab |
| 1fqi | 1g5a | 1gak |
| 1fvi | 1g66 | 1gal |
| 1fyj | 1g7r | 1gd4 |
| 1g03 | 1gc6 | 1gdf |
| 1g2r | 1gcf | 1gh8 |
| 1g6e | 1gcu | 1gh9 |
| 1g7e | 1gd5 | 1grj |
| 1g9o | 1ggw | 1gyf |
| 1ga3 | 1ghh | 1h6o |
| 1gcb | 1gnc | 1h6q |
| 1ge9 | 1gvp | 1h9e |
| 1ght | 1gwz | 1ha8 |
| 1glv | 1h4u | 1ha9 |
| 1gmx | 1h67 | 1hdo |
| 1gpi | 1h75 | 1hoe |
| 1gps | 1h8c | 1hu3 |
| 1h5p | 1hd2 | 1hy8 |
| 1h6h | 1hdp | 1hyb |
| 1hce | 1he9 | 1hyp |
| 1hdk | 1hg7 | 1hz4 |
| 1hdl | 1hnk | 1hzi |
| 1hfi | 1hns | 1i1s |
| 1hp2 | 1hs7 | 1i2t |
| 1hvb | 1hv6 | 1ica |
| 1hyk | 1hx2 | 1ie5 |
| 1hyw | 1hy9 | 1ie9 |
| 1i26 | 1hzl | 1igl |
| 1i9y | 1hzt | 1iio |
| 1ian | 1i17 | 1in5 |
| 1ig6 | 1i2v | 1itp |
| 1imt | 1i35 | 1ixh |
| 1iqq | 1i4w | 1jdn |
| 1j7q | 1ifc | 1jej |
| 1j8y | 1ija | 1jf8 |
| 1jbe | 1ipg | 1jgs |
| 1jce | 1irl | 1jqr |
| 1jei | 1itf | 1jt8 |
| 1ji6 | 1j8k | 1jw2 |
| 1ji8 | 1j9b | 1jw3 |
| 1jl1 | 1jbi | 1jyk |
| 1jrm | 1jeo | 1k8m |
| 1k0k | 1jik | 1kgd |
| 1k0s | 1jjr | 1krq |
| 1khc | 1jkz | 1krs |
| 1khm | 1jli | 1ksr |
| 1kot | 1juk | 1kul |
| 1kp6 | 1jwe | 1lip |
| 1kq8 | 1jyt | 1lkf |
| 1lfb | 1k7c | 1lre |
| 1mbj | 1k92 | 1mkn |
| 1mgt | 1k99 | 1mrj |
| 1moq | 1kgs | 1nar |
| 1mug | 1kik | 1neb |
| 1mut | 1kpf | 1ngl |
| 1mwp | 1ks0 | 1ngr |
| 1ner | 1kte | 1oaa |
| 1nkd | 1kth | 1pdy |
| 1nkr | 1mai | 1qau |
| 1opd | 1mkc | 1qcz |
| 1orc | 1mla | 1qdd |
| 1pba | 1mml | 1qgm |
| 1pht | 1nkl | 1qgv |
| 1pou | 1nox | 1qhk |
| 1ppn | 1nuk | 1qk7 |
| 1qdp | 1nxb | 1qmt |
| 1qhl | 1pdo | 1qp3 |
| 1qj4 | 1pfo | 1qqf |
| 1qkf | 1pgs | 1qst |
| 1qld | 1qk6 | 1qu5 |
| 1qpv | 1qky | 1res |
| 1qtf | 1qml | 1rfa |
| 1qts | 1qnr | 1rgs |
| 1rhs | 1qqi | 1roo |
| 1rl6 | 1qqq | 1sfp |
| 1rss | 1qto | 1t27 |
| 1sis | 1scy | 1tia |
| 1t1d | 1tig | 1tl2 |
| 1tfb | 1tml | 1tle |
| 1tul | 1tpn | 1ttg |
| 1utg | 1u2f | 1vdc |
| 1vcc | 1vib | 1vr2 |
| 1vie | 1vig | 1whi |
| 1vii | 1vls | 1wkt |
| 2acy | 1xbl | 1ysc |
| 2adx | 1xnt | 1yub |
| 2aw0 | 2af8 | 1zaq |
| 2bb8 | 2bby | 1zug |
| 2bds | 2cbh | 2bid |
| 2ezl | 2erl | 2brz |
| 2igd | 2fmr | 2gp8 |
| 2ila | 2fsp | 2hgf |
| 2jv3 | 2hsp | 2mst |
| 2lis | 2jdx | 2pnb |
| 2nef | 2myo | 2sh1 |
| 2nlr | 2sak | 2sn3 |
| 2pth | 2tbd | 2vgh |
| 2rgf | 3crd | 3lzt |
| 2sxl | 3gcc | 3sil |
| 3bbg | 3pyp | 3vub |
| 3ncm | 3tdt | 4eug |
| 3tss | 7a3h | 8abp |
| 8ohm |  |  |
