# Supplementary material for: Prediction of Protein Binding Regions in Disordered Proteins
Source: PLoS Comput Biol. 2009 May 1;5(5):e1000376. doi: 10.1371/journal.pcbi.1000376 (PMC2671142; doi:10.1371/journal.pcbi.1000376)
Supplement: Dataset S4 — 72 complexes of ordered proteins [3]. The interaction is considered between the shortest chains and its interaction partners. (0.08 MB DOC) [file pcbi.1000376.s004.doc]

| PDB ID | Shortest chain | Interacting partner(s) | PDB ID | Shortest chain | Interacting partner(s) |
| --- | --- | --- | --- | --- | --- |
| 1a0o | B | A | 1tar | A | B |
| 1acb | I | E | 1tgs | I | Z |
| 1ahw | C | AB | 1ttq | A | B |
| 1aoz | A | B | 1ugh | I | E |
| 1atn | D | A | 1utg | A | B |
| 1avw | B | A | 1vsg | A | B |
| 1avz | C | AB | 1ypi | A | B |
| 1brs | D | A | 1yqv | Y | LH |
| 1cdt | A | B | 2btf | P | A |
| 1cho | I | E | 2ccy | A | B |
| 1cse | I | E | 2cts | A | B |
| 1dfj | E | I | 2gn5 | A | B |
| 1fc1 | A | B | 2jel | P | LH |
| 1fdl | Y | LH | 2or1 | L | R |
| 1fss | B | A | 2pcb | B | AC |
| 1g6n | A | B | 2ptc | I | E |
| 1glb | F | G | 2rhe | A | B |
| 1glq | A | B | 2rus | A | B |
| 1hng | A | B | 2rve | A | B |
| 1hrp | A | B | 2sic | I | E |
| 1iai | MI | LH | 2sod | O | Y |
| 1il8 | A | B | 2ts1 | A | B |
| 1jhl | A | LH | 2tsc | A | B |
| 1lpa | A | B | 2vir | C | AB |
| 1luc | B | A | 3enl | A | B |
| 1mct | I | A | 3grs | A | B |
| 1mel | L | A | 3hfm | Y | LH |
| 1msb | A | B | 3hhr | A | BC |
| 1nca | N | LH | 3hvt | B | A |
| 1nl3 | A | B | 3icd | A | B |
| 1phh | A | B | 3sdh | A | B |
| 1pp2 | R | L | 3sdp | A | B |
| 1pyp | A | B | 3ssi | A | B |
| 1spb | P | S | 4htc | I | LH |
| 1stf | I | E | 4mdh | A | B |
| 1tab | I | E | 5adh | A | B |
