# Supplementary material for: Prediction of Protein Binding Regions in Disordered Proteins
Source: PLoS Comput Biol. 2009 May 1;5(5):e1000376. doi: 10.1371/journal.pcbi.1000376 (PMC2671142; doi:10.1371/journal.pcbi.1000376)
Supplement: Dataset S5 — The 53 complete archaea proteomes available from SwissProt (ftp://ftp.expasy.org/) used for full proteome scans. The fraction of total amino acids in disordered regions and the fraction of disordered amino acids in disordered binding sites are indicated together for each organism. (0.09 MB DOC) [file pcbi.1000376.s005.doc]

| SwissProt ID | Organism name | Fraction of amino acids in disordered regions | Fraction of disordered amino acids in binding regions |
| --- | --- | --- | --- |
| AERPE | Aeropyrum pernix | 0.0323 | 0.3262 |
| ARCFU | Archaeoglobus fulgidus | 0.0149 | 0.4539 |
| CALMQ | Caldivirga maquilingensis | 0.0144 | 0.3191 |
| CENSY | Cenarchaeum symbiosum | 0.1253 | 0.5189 |
| HALMA | Haloarcula marismortui | 0.2378 | 0.5515 |
| HALS3 | Halobacterium salinarum | 0.2111 | 0.5176 |
| HALSA | Halobacterium salinarum | 0.2166 | 0.5216 |
| HALWD | Haloquadratum walsbyi | 0.2234 | 0.5218 |
| HYPBU | Hyperthermus butylicus | 0.0238 | 0.3181 |
| IGNH4 | Ignicoccus hospitalis | 0.0253 | 0.3912 |
| KORCO | Korarchaeum cryptofilum | 0.0174 | 0.3347 |
| META3 | Methanococcus aeolicus | 0.0257 | 0.5278 |
| METAC | Methanosarcina acetivorans | 0.0554 | 0.5207 |
| METB6 | Methanoregula boonei | 0.0561 | 0.4426 |
| METBF | Methanosarcina barkeri | 0.0533 | 0.5021 |
| METBU | Methanococcoides burtonii | 0.0389 | 0.4586 |
| METHJ | Methanospirillum hungatei | 0.0482 | 0.4868 |
| METJA | Methanocaldococcus jannaschii | 0.0111 | 0.4911 |
| METKA | Methanopyrus kandleri | 0.0725 | 0.4324 |
| METLZ | Methanocorpusculum labreanum | 0.0339 | 0.4335 |
| METM5 | Methanococcus maripaludis | 0.0225 | 0.4792 |
| METM6 | Methanococcus maripaludis | 0.0229 | 0.5025 |
| METM7 | Methanococcus maripaludis | 0.0233 | 0.5010 |
| METMA | Methanosarcina mazei | 0.0529 | 0.4986 |
| METMJ | Methanoculleus marisnigri | 0.0605 | 0.4713 |
| METMP | Methanococcus maripaludis | 0.0225 | 0.4994 |
| METS3 | Methanobrevibacter smithii | 0.0351 | 0.4977 |
| METS5 | Metallosphaera sedula | 0.0220 | 0.3476 |
| METST | Methanosphaera stadtmanae | 0.0486 | 0.4723 |
| METTH | Methanobacterium thermoautotrophicum | 0.0428 | 0.4326 |
| METTP | Methanosaeta thermophila | 0.0380 | 0.4035 |
| METVS | Methanococcus vannielii | 0.0170 | 0.4633 |
| NANEQ | Nanoarchaeum equitans | 0.0126 | 0.5076 |
| NATPD | Natronomonas pharaonis | 0.2391 | 0.5661 |
| NITMS | Nitrosopumilus maritimus | 0.0685 | 0.5039 |
| PICTO | Picrophilus torridus | 0.0135 | 0.4314 |
| PYRAB | Pyrococcus abyssi | 0.0144 | 0.3555 |
| PYRAE | Pyrobaculum aerophilum | 0.0179 | 0.3731 |
| PYRAR | Pyrobaculum arsenaticum | 0.0182 | 0.3495 |
| PYRCJ | Pyrobaculum calidifontis | 0.0180 | 0.3674 |
| PYRFU | Pyrococcus furiosus | 0.0139 | 0.3667 |
| PYRHO | Pyrococcus horikoshii | 0.0154 | 0.3698 |
| PYRIL | Pyrobaculum islandicum | 0.0181 | 0.3718 |
| PYRKO | Pyrococcus kodakaraensis | 0.0241 | 0.4001 |
| STAMF | Staphylothermus marinus | 0.0130 | 0.3710 |
| SULAC | Sulfolobus acidocaldarius | 0.0131 | 0.3612 |
| SULSO | Sulfolobus solfataricus | 0.0110 | 0.4006 |
| SULTO | Sulfolobus tokodaii | 0.0117 | 0.3993 |
| THEAC | Thermoplasma acidophilum | 0.0214 | 0.3592 |
| THENV | Thermoproteus neutrophilus | 0.0214 | 0.3413 |
| THEPD | Thermofilum pendens | 0.0194 | 0.3523 |
| THEVO | Thermoplasma volcanium | 0.0199 | 0.4035 |
| UNCMA | Uncultured methanogenic archaeon RC-I | 0.0524 | 0.4517 |
