# Supplementary material for: Prediction of Protein Binding Regions in Disordered Proteins
Source: PLoS Comput Biol. 2009 May 1;5(5):e1000376. doi: 10.1371/journal.pcbi.1000376 (PMC2671142; doi:10.1371/journal.pcbi.1000376)
Supplement: Dataset S6 — The 639 complete bacteria proteomes available from SwissProt (ftp://ftp.expasy.org/) used for full proteome scans. The fraction of total amino acids in disordered regions and the fraction of disordered amino acids in disordered binding sites are indicated together for each organism. (0.86 MB DOC) [file pcbi.1000376.s006.doc]

| SwissProt ID | Organism name | Fraction of amino acids in disordered regions | Fraction of disordered amino acids in binding regions |
| --- | --- | --- | --- |
| ACAM1 | Acaryochloris marina | 0.0863 | 0.5018 |
| ACHLI | Acholeplasma laidlawii | 0.0186 | 0.4057 |
| ACIAC | Acidovorax avenae subsp. citrulli | 0.1221 | 0.4840 |
| ACIAD | Acinetobacter sp | 0.0469 | 0.4178 |
| ACIBC | Acinetobacter baumannii | 0.0513 | 0.4301 |
| ACIBL | Acidobacteria bacterium | 0.0887 | 0.4614 |
| ACIBT | Acinetobacter baumannii | 0.0504 | 0.4347 |
| ACIBY | Acinetobacter baumannii | 0.0514 | 0.4471 |
| ACIC1 | Acidothermus cellulolyticus | 0.1102 | 0.4551 |
| ACICJ | Acidiphilium cryptum | 0.0779 | 0.4132 |
| ACISJ | Acidovorax sp | 0.0951 | 0.4480 |
| ACTP2 | Actinobacillus pleuropneumoniae serotype | 0.0456 | 0.4103 |
| ACTP7 | Actinobacillus pleuropneumoniae serotype | 0.0471 | 0.3991 |
| ACTPJ | Actinobacillus pleuropneumoniae serotype | 0.0404 | 0.4248 |
| ACTSZ | Actinobacillus succinogenes | 0.0437 | 0.4085 |
| AERHH | Aeromonas hydrophila subsp. hydrophila | 0.0556 | 0.4121 |
| AERS4 | Aeromonas salmonicida | 0.0577 | 0.4238 |
| AGRT5 | Agrobacterium tumefaciens | 0.0693 | 0.4271 |
| AKKM8 | Akkermansia muciniphila | 0.0794 | 0.4872 |
| ALCBS | Alcanivorax borkumensis | 0.0788 | 0.4510 |
| ALHEH | Alkalilimnicola ehrlichei | 0.1180 | 0.4559 |
| ALKMQ | Alkaliphilus metalliredigens | 0.0361 | 0.4580 |
| ALKOO | Alkaliphilus oremlandii | 0.0281 | 0.4555 |
| AMOA5 | Amoebophilus asiaticus | 0.0621 | 0.5090 |
| ANADE | Anaeromyxobacter dehalogenans | 0.1189 | 0.4631 |
| ANADF | Anaeromyxobacter sp | 0.1209 | 0.4658 |
| ANAMM | Anaplasma marginale | 0.0785 | 0.5966 |
| ANAPZ | Anaplasma phagocytophilum | 0.0595 | 0.5708 |
| ANASP | Anabaena sp | 0.0622 | 0.4556 |
| ANAVT | Anabaena variabilis | 0.0614 | 0.4484 |
| AQUAE | Aquifex aeolicus | 0.0162 | 0.4531 |
| ARCB4 | Arcobacter butzleri | 0.0275 | 0.4788 |
| ARTAT | Arthrobacter aurescens | 0.1203 | 0.4598 |
| ARTS2 | Arthrobacter sp | 0.1218 | 0.4554 |
| AYWBP | Aster yellows witches'-broom phytoplasma | 0.0510 | 0.5190 |
| AZOC5 | Azorhizobium caulinodans | 0.0845 | 0.4433 |
| AZOSB | Azoarcus sp | 0.0692 | 0.4117 |
| AZOSE | Azoarcus sp | 0.0905 | 0.4857 |
| BACA2 | Bacillus amyloliquefaciens | 0.0590 | 0.4570 |
| BACAH | Bacillus thuringiensis | 0.0460 | 0.4616 |
| BACAN | Bacillus anthracis | 0.0443 | 0.4595 |
| BACC1 | Bacillus cereus | 0.0474 | 0.4818 |
| BACCN | Bacillus cereus subsp. cytotoxis | 0.0446 | 0.4638 |
| BACCR | Bacillus cereus | 0.0465 | 0.4622 |
| BACCZ | Bacillus cereus | 0.0453 | 0.4552 |
| BACFN | Bacteroides fragilis | 0.0333 | 0.5149 |
| BACFR | Bacteroides fragilis | 0.0339 | 0.5237 |
| BACHD | Bacillus halodurans | 0.0561 | 0.4718 |
| BACHK | Bacillus thuringiensis subsp. konkukian | 0.0460 | 0.4526 |
| BACLD | Bacillus licheniformis | 0.0581 | 0.4613 |
| BACP2 | Bacillus pumilus | 0.0585 | 0.4350 |
| BACSK | Bacillus clausii | 0.0592 | 0.4844 |
| BACSU | Bacillus subtilis | 0.0571 | 0.4583 |
| BACTN | Bacteroides thetaiotaomicron | 0.0348 | 0.5352 |
| BACV8 | Bacteroides vulgatus | 0.0354 | 0.5189 |
| BACWK | Bacillus weihenstephanensis | 0.0477 | 0.4637 |
| BARBK | Bartonella bacilliformis | 0.0523 | 0.4516 |
| BARHE | Bartonella henselae | 0.0633 | 0.4840 |
| BARQU | Bartonella quintana | 0.0529 | 0.4690 |
| BART1 | Bartonella tribocorum | 0.0719 | 0.4721 |
| BAUCH | Baumannia cicadellinicola subsp. Homalod | 0.0167 | 0.4591 |
| BDEBA | Bdellovibrio bacteriovorus | 0.0634 | 0.4640 |
| BEII9 | Beijerinckia indica subsp. indica | 0.0760 | 0.4428 |
| BIFAA | Bifidobacterium adolescentis | 0.1284 | 0.4969 |
| BIFLD | Bifidobacterium longum | 0.1308 | 0.4792 |
| BIFLO | Bifidobacterium longum | 0.1337 | 0.4910 |
| BLOFL | Blochmannia floridanus | 0.0137 | 0.5537 |
| BLOPB | Blochmannia pennsylvanicus | 0.0181 | 0.4828 |
| BORA1 | Bordetella avium | 0.0700 | 0.4030 |
| BORAP | Borrelia afzelii | 0.0235 | 0.5614 |
| BORBR | Bordetella bronchiseptica | 0.0719 | 0.4111 |
| BORBU | Borrelia burgdorferi | 0.0327 | 0.5452 |
| BORGA | Borrelia garinii | 0.0247 | 0.5521 |
| BORHD | Borrelia hermsii | 0.0195 | 0.4574 |
| BORPA | Bordetella parapertussis | 0.0704 | 0.4087 |
| BORPD | Bordetella petrii | 0.0790 | 0.4293 |
| BORPE | Bordetella pertussis | 0.0702 | 0.4012 |
| BRAJA | Bradyrhizobium japonicum | 0.0892 | 0.4580 |
| BRASB | Bradyrhizobium sp | 0.0829 | 0.4520 |
| BRASO | Bradyrhizobium sp | 0.0882 | 0.4612 |
| BRUA2 | Brucella abortus | 0.0734 | 0.4441 |
| BRUAB | Brucella abortus | 0.0739 | 0.4442 |
| BRUC2 | Brucella canis | 0.0735 | 0.4465 |
| BRUME | Brucella melitensis | 0.0717 | 0.4275 |
| BRUO2 | Brucella ovis | 0.0744 | 0.4456 |
| BRUSI | Brucella suis | 0.0745 | 0.4476 |
| BRUSU | Brucella suis | 0.0726 | 0.4441 |
| BUCAI | Buchnera aphidicola subsp. Acyrthosiphon | 0.0189 | 0.5622 |
| BUCAP | Buchnera aphidicola subsp. Schizaphis gr | 0.0170 | 0.5841 |
| BUCBP | Buchnera aphidicola subsp. Baizongia pis | 0.0155 | 0.5773 |
| BUCCC | Buchnera aphidicola subsp. Cinara cedri | 0.0118 | 0.6714 |
| BURA4 | Burkholderia ambifaria | 0.0798 | 0.4322 |
| BURCA | Burkholderia cenocepacia | 0.0785 | 0.4372 |
| BURCC | Burkholderia cenocepacia | 0.0748 | 0.4169 |
| BURCH | Burkholderia cenocepacia | 0.0777 | 0.4337 |
| BURCM | Burkholderia ambifaria | 0.0812 | 0.4358 |
| BURM1 | Burkholderia multivorans | 0.0813 | 0.4422 |
| BURM7 | Burkholderia mallei | 0.1111 | 0.5245 |
| BURM9 | Burkholderia mallei | 0.1054 | 0.5134 |
| BURMA | Burkholderia mallei | 0.1052 | 0.5158 |
| BURMS | Burkholderia mallei | 0.1085 | 0.5204 |
| BURP0 | Burkholderia pseudomallei | 0.1145 | 0.5315 |
| BURP1 | Burkholderia pseudomallei | 0.1864 | 0.5479 |
| BURP6 | Burkholderia pseudomallei | 0.1146 | 0.5317 |
| BURP8 | Burkholderia phymatum | 0.0797 | 0.4346 |
| BURPP | Burkholderia phytofirmans | 0.0770 | 0.4340 |
| BURPS | Burkholderia pseudomallei | 0.0849 | 0.4398 |
| BURS3 | Burkholderia sp | 0.0769 | 0.4299 |
| BURTA | Burkholderia thailandensis | 0.0958 | 0.4716 |
| BURVG | Burkholderia vietnamiensis | 0.0884 | 0.4634 |
| BURXL | Burkholderia xenovorans | 0.0796 | 0.4344 |
| CALS8 | Caldicellulosiruptor saccharolyticus | 0.0173 | 0.5015 |
| CAMC1 | Campylobacter concisus | 0.0387 | 0.4766 |
| CAMC5 | Campylobacter curvus | 0.0303 | 0.4551 |
| CAMFF | Campylobacter fetus subsp. fetus | 0.0239 | 0.4333 |
| CAMHC | Campylobacter hominis | 0.0263 | 0.4616 |
| CAMJ8 | Campylobacter jejuni subsp. jejuni serot | 0.0262 | 0.4812 |
| CAMJD | Campylobacter jejuni subsp. doylei | 0.0256 | 0.4593 |
| CAMJE | Campylobacter jejuni | 0.0259 | 0.4669 |
| CAMJJ | Campylobacter jejuni subsp. jejuni serot | 0.0287 | 0.4813 |
| CAMJR | Campylobacter jejuni | 0.0276 | 0.4766 |
| CARHZ | Carboxydothermus hydrogenoformans | 0.0203 | 0.3944 |
| CARRP | Carsonella ruddii | 0.0046 | 0.9348 |
| CAUCR | Caulobacter crescentus | 0.0947 | 0.4521 |
| CAUSK | Caulobacter sp | 0.0838 | 0.4133 |
| CHLAA | Chloroflexus aurantiacus | 0.0623 | 0.4318 |
| CHLAB | Chlamydophila abortus | 0.0525 | 0.4895 |
| CHLCH | Chlorobium chlorochromatii | 0.0486 | 0.4070 |
| CHLCV | Chlamydophila caviae | 0.0542 | 0.5012 |
| CHLFF | Chlamydophila felis | 0.0524 | 0.5130 |
| CHLL2 | Chlorobium limicola | 0.0571 | 0.4767 |
| CHLMU | Chlamydia muridarum | 0.0457 | 0.5168 |
| CHLPB | Chlorobium phaeobacteroides | 0.0513 | 0.4484 |
| CHLPD | Chlorobium phaeobacteroides | 0.0465 | 0.4409 |
| CHLPN | Chlamydia pneumoniae | 0.0503 | 0.5192 |
| CHLT2 | Chlamydia trachomatis | 0.0504 | 0.4990 |
| CHLTA | Chlamydia trachomatis | 0.0518 | 0.5086 |
| CHLTB | Chlamydia trachomatis | 0.0503 | 0.4986 |
| CHLTE | Chlorobium tepidum | 0.0512 | 0.4372 |
| CHLTR | Chlamydia trachomatis | 0.0523 | 0.5112 |
| CHRSD | Chromohalobacter salexigens | 0.0954 | 0.4401 |
| CHRVO | Chromobacterium violaceum | 0.0743 | 0.4594 |
| CITK8 | Citrobacter koseri | 0.0556 | 0.4388 |
| CLAM3 | Clavibacter michiganensis subsp. michiga | 0.1404 | 0.4461 |
| CLAMS | Clavibacter michiganensis subsp. sepedon | 0.1500 | 0.4656 |
| CLOAB | Clostridium acetobutylicum | 0.0277 | 0.4879 |
| CLOB1 | Clostridium botulinum | 0.0262 | 0.5255 |
| CLOB8 | Clostridium beijerinckii | 0.0307 | 0.4777 |
| CLOBA | Clostridium botulinum | 0.0267 | 0.5027 |
| CLOBB | Clostridium botulinum | 0.0276 | 0.5067 |
| CLOBH | Clostridium botulinum | 0.0257 | 0.5309 |
| CLOBK | Clostridium botulinum | 0.0262 | 0.5281 |
| CLOBL | Clostridium botulinum | 0.0266 | 0.5249 |
| CLOBM | Clostridium botulinum | 0.0265 | 0.5241 |
| CLOD6 | Clostridium difficile | 0.0286 | 0.5234 |
| CLOK5 | Clostridium kluyveri | 0.0290 | 0.4791 |
| CLONN | Clostridium novyi | 0.0290 | 0.4779 |
| CLOP1 | Clostridium perfringens | 0.0329 | 0.5171 |
| CLOPE | Clostridium perfringens | 0.0310 | 0.5198 |
| CLOPH | Clostridium phytofermentans | 0.0342 | 0.4966 |
| CLOPS | Clostridium perfringens | 0.0283 | 0.5326 |
| CLOTE | Clostridium tetani | 0.0225 | 0.5104 |
| CLOTH | Clostridium thermocellum | 0.0359 | 0.5026 |
| COLP3 | Colwellia psychrerythraea | 0.0440 | 0.4230 |
| CORDI | Corynebacterium diphtheriae | 0.1049 | 0.4531 |
| COREF | Corynebacterium efficiens | 0.1392 | 0.4935 |
| CORGB | Corynebacterium glutamicum | 0.1037 | 0.4523 |
| CORGL | Corynebacterium glutamicum | 0.1073 | 0.4622 |
| CORJK | Corynebacterium jeikeium | 0.1769 | 0.5026 |
| COXBN | Coxiella burnetii | 0.0476 | 0.4778 |
| COXBR | Coxiella burnetii | 0.0479 | 0.4802 |
| COXBU | Coxiella burnetii | 0.0480 | 0.4847 |
| CUPTR | Cupriavidus taiwanensis | 0.0854 | 0.4227 |
| CYAA5 | Cyanothece | 0.0645 | 0.4878 |
| CYTH3 | Cytophaga hutchinsonii | 0.0282 | 0.4805 |
| DECAR | Dechloromonas aromatica | 0.0626 | 0.4254 |
| DEHE1 | Dehalococcoides ethenogenes | 0.0416 | 0.4202 |
| DEHSB | Dehalococcoides sp | 0.0412 | 0.4030 |
| DEHSC | Dehalococcoides sp | 0.0381 | 0.4050 |
| DEIGD | Deinococcus geothermalis | 0.1046 | 0.4346 |
| DEIRA | Deinococcus radiodurans | 0.1319 | 0.4816 |
| DELAS | Delftia acidovorans | 0.0968 | 0.4427 |
| DESAP | Desulforudis audaxviator | 0.0493 | 0.3937 |
| DESDG | Desulfovibrio desulfuricans | 0.0737 | 0.4782 |
| DESHY | Desulfitobacterium hafniense | 0.0436 | 0.4632 |
| DESOH | Desulfococcus oleovorans | 0.0524 | 0.4552 |
| DESPS | Desulfotalea psychrophila | 0.0413 | 0.4471 |
| DESRM | Desulfotomaculum reducens | 0.0413 | 0.4351 |
| DESVH | Desulfovibrio vulgaris | 0.0933 | 0.5045 |
| DESVV | Desulfovibrio vulgaris subsp. vulgaris | 0.0889 | 0.4964 |
| DICNV | Dichelobacter nodosus | 0.0533 | 0.4412 |
| DINSH | Dinoroseobacter shibae | 0.0851 | 0.4479 |
| ECO24 | Escherichia coli O139:H28 | 0.0561 | 0.4324 |
| ECO57 | Escherichia coli O157:H7 | 0.0646 | 0.4513 |
| ECODH | Escherichia coli | 0.0515 | 0.4202 |
| ECOHS | Escherichia coli O9:H4 | 0.0526 | 0.4227 |
| ECOK1 | Escherichia coli O1:K1 / APEC | 0.0573 | 0.4352 |
| ECOL5 | Escherichia coli O6:K15:H31 | 0.0518 | 0.4257 |
| ECOL6 | Escherichia coli O6 | 0.0549 | 0.4361 |
| ECOLC | Escherichia coli | 0.0539 | 0.4186 |
| ECOLI | Escherichia coli | 0.0533 | 0.4219 |
| ECOSM | Escherichia coli | 0.0541 | 0.4282 |
| ECOUT | Escherichia coli | 0.0564 | 0.4404 |
| EHRCJ | Ehrlichia canis | 0.0523 | 0.5550 |
| EHRCR | Ehrlichia chaffeensis | 0.0495 | 0.5809 |
| EHRRG | Ehrlichia ruminantium | 0.0521 | 0.5614 |
| EHRRW | Ehrlichia ruminantium | 0.0545 | 0.5755 |
| ELUMP | Elusimicrobium minutum | 0.0400 | 0.4817 |
| ENT38 | Enterobacter sp | 0.0589 | 0.4235 |
| ENTFA | Enterococcus faecalis | 0.0666 | 0.4860 |
| ENTS8 | Enterobacter sakazakii | 0.0623 | 0.4376 |
| ERWCT | Erwinia carotovora subsp. atroseptica | 0.0607 | 0.4075 |
| ERYLH | Erythrobacter litoralis | 0.1105 | 0.4752 |
| EXIS2 | Exiguobacterium sibiricum | 0.0500 | 0.4237 |
| FERNB | Fervidobacterium nodosum | 0.0159 | 0.3728 |
| FINM2 | Finegoldia magna | 0.0525 | 0.4943 |
| FLAJO | Flavobacterium johnsoniae | 0.0317 | 0.5179 |
| FLAPJ | Flavobacterium psychrophilum | 0.0290 | 0.4581 |
| FRAAA | Frankia alni | 0.1918 | 0.5632 |
| FRAP2 | Francisella philomiragia subsp. philomir | 0.0262 | 0.4897 |
| FRASC | Frankia sp | 0.1761 | 0.5405 |
| FRASN | Frankia sp | 0.1823 | 0.5496 |
| FRAT1 | Francisella tularensis subsp. tularensis | 0.0258 | 0.5008 |
| FRATF | Francisella tularensis subsp. holarctica | 0.0249 | 0.4963 |
| FRATH | Francisella tularensis subsp. holarctica | 0.0246 | 0.4968 |
| FRATM | Francisella tularensis subsp. mediasiati | 0.0265 | 0.4984 |
| FRATN | Francisella tularensis subsp. novicida | 0.0257 | 0.4995 |
| FRATO | Francisella tularensis subsp. holarctica | 0.0254 | 0.5014 |
| FRATT | Francisella tularensis subsp. tularensis | 0.0257 | 0.5001 |
| FRATW | Francisella tularensis subsp. tularensis | 0.0254 | 0.4952 |
| FUSNN | Fusobacterium nucleatum subsp. nucleatum | 0.0243 | 0.4460 |
| GEOKA | Geobacillus kaustophilus | 0.0491 | 0.4553 |
| GEOLS | Geobacter lovleyi | 0.0498 | 0.4406 |
| GEOMG | Geobacter metallireducens | 0.0565 | 0.4325 |
| GEOSL | Geobacter sulfurreducens | 0.0594 | 0.4474 |
| GEOTN | Geobacillus thermodenitrificans | 0.0455 | 0.4558 |
| GEOUR | Geobacter uraniireducens | 0.0470 | 0.4079 |
| GLOVI | Gloeobacter violaceus | 0.0766 | 0.4609 |
| GLUDA | Gluconacetobacter diazotrophicus | 0.1064 | 0.4596 |
| GLUOX | Gluconobacter oxydans | 0.1092 | 0.4732 |
| GRABC | Granulibacter bethesdensis | 0.0932 | 0.4513 |
| GRAFK | Gramella forsetii | 0.0522 | 0.5402 |
| HAEDU | Haemophilus ducreyi | 0.0467 | 0.4686 |
| HAEI8 | Haemophilus influenzae | 0.0464 | 0.4422 |
| HAEIE | Haemophilus influenzae | 0.0411 | 0.4393 |
| HAEIG | Haemophilus influenzae | 0.0417 | 0.4443 |
| HAEIN | Haemophilus influenzae | 0.0419 | 0.4500 |
| HAES1 | Haemophilus somnus | 0.0576 | 0.4094 |
| HAES2 | Haemophilus somnus | 0.0590 | 0.4133 |
| HAHCH | Hahella chejuensis | 0.0677 | 0.4593 |
| HALHL | Halorhodospira halophila | 0.1462 | 0.4738 |
| HELAH | Helicobacter acinonychis | 0.0397 | 0.4880 |
| HELHP | Helicobacter hepaticus | 0.0312 | 0.4942 |
| HELMI | Heliobacterium modesticaldum | 0.0691 | 0.4437 |
| HELPH | Helicobacter pylori | 0.0458 | 0.4957 |
| HELPJ | Helicobacter pylori J99 | 0.0476 | 0.4936 |
| HELPS | Helicobacter pylori | 0.0456 | 0.5065 |
| HELPY | Helicobacter pylori | 0.0459 | 0.5072 |
| HERA2 | Herpetosiphon aurantiacus | 0.0646 | 0.4596 |
| HERAR | Herminiimonas arsenicoxydans | 0.0641 | 0.4261 |
| HYPNA | Hyphomonas neptunium | 0.0869 | 0.4420 |
| IDILO | Idiomarina loihiensis | 0.0750 | 0.4497 |
| JANMA | Janthinobacterium sp | 0.0619 | 0.3991 |
| JANSC | Jannaschia sp | 0.0847 | 0.4444 |
| KINRD | Kineococcus radiotolerans | 0.1643 | 0.4904 |
| KLEP7 | Klebsiella pneumoniae subsp. pneumoniae | 0.0527 | 0.4151 |
| KOCRD | Kocuria rhizophila | 0.1932 | 0.5309 |
| LACAC | Lactobacillus acidophilus | 0.0756 | 0.4735 |
| LACBA | Lactobacillus brevis | 0.0698 | 0.4130 |
| LACC3 | Lactobacillus casei | 0.0683 | 0.4344 |
| LACDA | Lactobacillus delbrueckii subsp. bulgari | 0.0527 | 0.4361 |
| LACDB | Lactobacillus delbrueckii subsp. bulgari | 0.0521 | 0.4390 |
| LACF3 | Lactobacillus fermentum | 0.0665 | 0.4188 |
| LACGA | Lactobacillus gasseri | 0.0718 | 0.4505 |
| LACH4 | Lactobacillus helveticus | 0.0542 | 0.4487 |
| LACJO | Lactobacillus johnsonii | 0.0813 | 0.4308 |
| LACLA | Lactococcus lactis subsp. lactis | 0.0489 | 0.4063 |
| LACLM | Lactococcus lactis subsp. cremoris | 0.0496 | 0.4222 |
| LACLS | Lactococcus lactis subsp. cremoris | 0.0474 | 0.4167 |
| LACPL | Lactobacillus plantarum | 0.0657 | 0.4095 |
| LACRF | Lactobacillus reuteri | 0.0738 | 0.4433 |
| LACS1 | Lactobacillus salivarius subsp. salivari | 0.0530 | 0.4326 |
| LACSS | Lactobacillus sakei subsp. sakei | 0.0606 | 0.4168 |
| LAWIP | Lawsonia intracellularis | 0.0639 | 0.5176 |
| LEGPA | Legionella pneumophila | 0.0427 | 0.4417 |
| LEGPC | Legionella pneumophila | 0.0438 | 0.4613 |
| LEGPH | Legionella pneumophila subsp. pneumophil | 0.0402 | 0.4561 |
| LEGPL | Legionella pneumophila | 0.0413 | 0.4487 |
| LEIXX | Leifsonia xyli subsp. xyli | 0.1110 | 0.4462 |
| LEPBA | Leptospira biflexa serovar Patoc | 0.0374 | 0.4943 |
| LEPBJ | Leptospira borgpetersenii serovar Hardjo | 0.0385 | 0.4885 |
| LEPBL | Leptospira borgpetersenii serovar Hardjo | 0.0383 | 0.4905 |
| LEPBP | Leptospira biflexa serovar Patoc | 0.0378 | 0.4907 |
| LEPCP | Leptothrix cholodnii | 0.0825 | 0.4172 |
| LEPIC | Leptospira interrogans serogroup Icteroh | 0.0377 | 0.4893 |
| LEPIN | Leptospira interrogans | 0.0374 | 0.5020 |
| LEUCK | Leuconostoc citreum | 0.0567 | 0.3870 |
| LEUMM | Leuconostoc mesenteroides subsp. mesente | 0.0576 | 0.3753 |
| LISIN | Listeria innocua | 0.0519 | 0.4428 |
| LISMF | Listeria monocytogenes serotype 4b | 0.0494 | 0.4521 |
| LISMO | Listeria monocytogenes | 0.0501 | 0.4625 |
| LISW6 | Listeria welshimeri serovar 6b | 0.0495 | 0.4642 |
| LYSSC | Lysinibacillus sphaericus | 0.0400 | 0.4443 |
| MAGMM | Magnetospirillum magneticum | 0.0920 | 0.4334 |
| MAGSM | Magnetococcus sp | 0.0919 | 0.4686 |
| MANSM | Mannheimia succiniciproducens | 0.0410 | 0.4093 |
| MARAV | Marinobacter aquaeolei | 0.0927 | 0.4616 |
| MARMM | Maricaulis maris | 0.0940 | 0.4336 |
| MARMS | Marinomonas sp | 0.0448 | 0.4090 |
| MESFL | Mesoplasma florum | 0.0294 | 0.4245 |
| MESSB | Mesorhizobium sp | 0.0833 | 0.4471 |
| METCA | Methylococcus capsulatus | 0.0764 | 0.4604 |
| METEP | Methylobacterium extorquens | 0.1127 | 0.4724 |
| METFK | Methylobacillus flagellatus | 0.0612 | 0.4201 |
| METI4 | Methylokorus infernorum | 0.0407 | 0.5021 |
| METPB | Methylobacterium populi | 0.1136 | 0.4722 |
| METPP | Methylibium petroleiphilum | 0.0856 | 0.4319 |
| METRJ | Methylobacterium radiotolerans | 0.1050 | 0.4592 |
| METS4 | Methylobacterium sp | 0.1039 | 0.4546 |
| MICAN | Microcystis aeruginosa | 0.0518 | 0.4804 |
| MOOTA | Moorella thermoacetica | 0.0454 | 0.4119 |
| MYCA1 | Mycobacterium avium | 0.1135 | 0.4645 |
| MYCAP | Mycoplasma agalactiae | 0.0442 | 0.5164 |
| MYCBO | Mycobacterium bovis | 0.1171 | 0.4246 |
| MYCBP | Mycobacterium bovis | 0.1176 | 0.4195 |
| MYCCT | Mycoplasma capricolum subsp. capricolum | 0.0431 | 0.5018 |
| MYCGA | Mycoplasma gallisepticum | 0.0831 | 0.5970 |
| MYCGE | Mycoplasma genitalium | 0.0486 | 0.5487 |
| MYCGI | Mycobacterium gilvum | 0.1233 | 0.4791 |
| MYCH2 | Mycoplasma hyopneumoniae | 0.0462 | 0.4917 |
| MYCH7 | Mycoplasma hyopneumoniae | 0.0438 | 0.4940 |
| MYCHJ | Mycoplasma hyopneumoniae | 0.0452 | 0.5010 |
| MYCLE | Mycobacterium leprae | 0.0887 | 0.4557 |
| MYCMM | Mycobacterium marinum | 0.1284 | 0.4043 |
| MYCMO | Mycoplasma mobile | 0.0245 | 0.4315 |
| MYCMS | Mycoplasma mycoides subsp. mycoides SC | 0.0361 | 0.5098 |
| MYCPA | Mycobacterium paratuberculosis | 0.1227 | 0.4789 |
| MYCPE | Mycoplasma penetrans | 0.0546 | 0.5769 |
| MYCPN | Mycoplasma pneumoniae | 0.0900 | 0.5333 |
| MYCPU | Mycoplasma pulmonis | 0.0558 | 0.5109 |
| MYCS2 | Mycobacterium smegmatis | 0.1119 | 0.4579 |
| MYCS5 | Mycoplasma synoviae | 0.0483 | 0.5220 |
| MYCSJ | Mycobacterium sp | 0.1175 | 0.4605 |
| MYCSK | Mycobacterium sp | 0.1232 | 0.4711 |
| MYCSS | Mycobacterium sp | 0.1227 | 0.4705 |
| MYCTA | Mycobacterium tuberculosis | 0.1158 | 0.4262 |
| MYCTU | Mycobacterium tuberculosis | 0.1176 | 0.4187 |
| MYCUA | Mycobacterium ulcerans | 0.1159 | 0.4538 |
| MYCVP | Mycobacterium vanbaalenii | 0.1166 | 0.4746 |
| MYXXD | Myxococcus xanthus | 0.1428 | 0.5268 |
| NATTJ | Natranaerobius thermophilus | 0.0884 | 0.5170 |
| NEIG1 | Neisseria gonorrhoeae | 0.0766 | 0.5048 |
| NEIM0 | Neisseria meningitidis serogroup C | 0.0729 | 0.4852 |
| NEIMA | Neisseria meningitidis serogroup A | 0.0699 | 0.4730 |
| NEIMB | Neisseria meningitidis serogroup B | 0.0782 | 0.4863 |
| NEIMF | Neisseria meningitidis serogroup C / ser | 0.0712 | 0.4650 |
| NEOSM | Neorickettsia sennetsu | 0.0389 | 0.5452 |
| NITEC | Nitrosomonas eutropha | 0.0577 | 0.4267 |
| NITEU | Nitrosomonas europaea | 0.0586 | 0.4326 |
| NITHX | Nitrobacter hamburgensis | 0.1032 | 0.4818 |
| NITMU | Nitrosospira multiformis | 0.0713 | 0.4358 |
| NITOC | Nitrosococcus oceani | 0.0637 | 0.4321 |
| NITSB | Nitratiruptor sp | 0.0218 | 0.4444 |
| NITWN | Nitrobacter winogradskyi | 0.1062 | 0.4887 |
| NOCFA | Nocardia farcinica | 0.1315 | 0.4845 |
| NOCSJ | Nocardioides sp | 0.1235 | 0.4644 |
| NOSP7 | Nostoc punctiforme | 0.0606 | 0.4436 |
| NOVAD | Novosphingobium aromaticivorans | 0.0795 | 0.4169 |
| OCEIH | Oceanobacillus iheyensis | 0.0625 | 0.4976 |
| OCHA4 | Ochrobactrum anthropi | 0.0738 | 0.4300 |
| OENOB | Oenococcus oeni | 0.0470 | 0.4308 |
| ONYPE | Onion yellows phytoplasma | 0.0608 | 0.5112 |
| OPITP | Opitutus terrae | 0.0775 | 0.4191 |
| ORITB | Orientia tsutsugamushi | 0.0357 | 0.5430 |
| ORITI | Orientia tsutsugamushi | 0.0380 | 0.5770 |
| PARD8 | Parabacteroides distasonis | 0.0322 | 0.5006 |
| PARDP | Paracoccus denitrificans | 0.0893 | 0.4419 |
| PARL1 | Parvibaculum lavamentivorans | 0.0822 | 0.4276 |
| PARUW | Protochlamydia amoebophila | 0.0384 | 0.4771 |
| PASMU | Pasteurella multocida | 0.0409 | 0.4137 |
| PEDPA | Pediococcus pentosaceus | 0.0663 | 0.3962 |
| PELCD | Pelobacter carbinolicus | 0.0533 | 0.4340 |
| PELLD | Pelodictyon luteolum | 0.0585 | 0.4074 |
| PELPD | Pelobacter propionicus | 0.0620 | 0.4657 |
| PELTS | Pelotomaculum thermopropionicum | 0.0417 | 0.4397 |
| PELUB | Pelagibacter ubique | 0.0362 | 0.4783 |
| PETMO | Petrotoga mobilis | 0.0216 | 0.4068 |
| PHOLL | Photorhabdus luminescens subsp. laumondi | 0.0564 | 0.4301 |
| PHOPR | Photobacterium profundum | 0.0498 | 0.4506 |
| POLNA | Polaromonas naphthalenivorans | 0.0743 | 0.4269 |
| POLNS | Polynucleobacter necessarius | 0.0548 | 0.4145 |
| POLSJ | Polaromonas sp | 0.0700 | 0.4122 |
| POLSQ | Polynucleobacter sp | 0.0510 | 0.4224 |
| PORG3 | Porphyromonas gingivalis | 0.0475 | 0.4972 |
| PORGI | Porphyromonas gingivalis | 0.0433 | 0.4844 |
| PROAC | Propionibacterium acnes | 0.1256 | 0.4769 |
| PROM0 | Prochlorococcus marinus | 0.0367 | 0.4654 |
| PROM1 | Prochlorococcus marinus | 0.0507 | 0.4665 |
| PROM2 | Prochlorococcus marinus | 0.0346 | 0.4818 |
| PROM3 | Prochlorococcus marinus | 0.0828 | 0.4753 |
| PROM4 | Prochlorococcus marinus | 0.0511 | 0.4523 |
| PROM5 | Prochlorococcus marinus | 0.0351 | 0.4615 |
| PROM9 | Prochlorococcus marinus | 0.0369 | 0.4811 |
| PROMA | Prochlorococcus marinus | 0.0486 | 0.4494 |
| PROMM | Prochlorococcus marinus | 0.0794 | 0.4540 |
| PROMP | Prochlorococcus marinus subsp. pastoris | 0.0348 | 0.4795 |
| PROMS | Prochlorococcus marinus | 0.0383 | 0.4557 |
| PROMT | Prochlorococcus marinus | 0.0527 | 0.4735 |
| PROVI | Prosthecochloris vibrioformis | 0.0586 | 0.4178 |
| PSE14 | Pseudomonas syringae pv. phaseolicola | 0.0702 | 0.4270 |
| PSEA6 | Pseudoalteromonas atlantica | 0.0579 | 0.4544 |
| PSEA7 | Pseudomonas aeruginosa | 0.0718 | 0.4371 |
| PSEAB | Pseudomonas aeruginosa | 0.0670 | 0.4151 |
| PSEAE | Pseudomonas aeruginosa | 0.0652 | 0.4143 |
| PSEE4 | Pseudomonas entomophila | 0.0655 | 0.4045 |
| PSEF5 | Pseudomonas fluorescens | 0.0631 | 0.4006 |
| PSEHT | Pseudoalteromonas haloplanktis | 0.0440 | 0.4363 |
| PSEMY | Pseudomonas mendocina | 0.0604 | 0.4026 |
| PSEP1 | Pseudomonas putida | 0.0665 | 0.4115 |
| PSEPF | Pseudomonas fluorescens | 0.0649 | 0.3969 |
| PSEPG | Pseudomonas putida | 0.0664 | 0.4087 |
| PSEPK | Pseudomonas putida | 0.0667 | 0.4144 |
| PSEPW | Pseudomonas putida | 0.0664 | 0.4085 |
| PSESM | Pseudomonas syringae pv. tomato | 0.0708 | 0.4329 |
| PSEU2 | Pseudomonas syringae pv. syringae | 0.0681 | 0.4207 |
| PSEU5 | Pseudomonas stutzeri | 0.0718 | 0.4479 |
| PSYA2 | Psychrobacter arcticus | 0.0826 | 0.4417 |
| PSYCK | Psychrobacter cryohalolentis | 0.0755 | 0.4449 |
| PSYIN | Psychromonas ingrahamii | 0.0356 | 0.4093 |
| PSYWF | Psychrobacter sp | 0.0996 | 0.4822 |
| RALEH | Ralstonia eutropha | 0.0779 | 0.4146 |
| RALEJ | Ralstonia eutropha | 0.0804 | 0.4214 |
| RALME | Ralstonia metallidurans | 0.0844 | 0.4304 |
| RALSO | Ralstonia solanacearum | 0.0986 | 0.4590 |
| RENSM | Renibacterium salmoninarum | 0.0944 | 0.4229 |
| RHIEC | Rhizobium etli | 0.0734 | 0.4371 |
| RHIL3 | Rhizobium leguminosarum bv. viciae | 0.0696 | 0.4323 |
| RHILO | Rhizobium loti | 0.0789 | 0.4333 |
| RHIME | Rhizobium meliloti | 0.0717 | 0.4238 |
| RHOBA | Rhodopirellula baltica | 0.1578 | 0.5369 |
| RHOFD | Rhodoferax ferrireducens | 0.0575 | 0.3940 |
| RHOP2 | Rhodopseudomonas palustris | 0.0851 | 0.4475 |
| RHOP5 | Rhodopseudomonas palustris | 0.0871 | 0.4528 |
| RHOPA | Rhodopseudomonas palustris | 0.0850 | 0.4620 |
| RHOPB | Rhodopseudomonas palustris | 0.0841 | 0.4483 |
| RHOPS | Rhodopseudomonas palustris | 0.0898 | 0.4624 |
| RHORT | Rhodospirillum rubrum | 0.0830 | 0.4255 |
| RHOS1 | Rhodobacter sphaeroides | 0.0864 | 0.4567 |
| RHOS4 | Rhodobacter sphaeroides | 0.0903 | 0.4613 |
| RHOS5 | Rhodobacter sphaeroides | 0.0915 | 0.4574 |
| RHOSR | Rhodococcus sp | 0.1339 | 0.4800 |
| RICAH | Rickettsia akari | 0.0404 | 0.4865 |
| RICB8 | Rickettsia bellii | 0.0451 | 0.5176 |
| RICBR | Rickettsia bellii | 0.0431 | 0.5008 |
| RICCK | Rickettsia canadensis | 0.0328 | 0.4852 |
| RICCN | Rickettsia conorii | 0.0363 | 0.4917 |
| RICFE | Rickettsia felis | 0.0461 | 0.4859 |
| RICM5 | Rickettsia massiliae | 0.0381 | 0.4661 |
| RICPR | Rickettsia prowazekii | 0.0248 | 0.4311 |
| RICRO | Rickettsia rickettsii | 0.0391 | 0.5080 |
| RICRS | Rickettsia rickettsii | 0.0401 | 0.5060 |
| RICTY | Rickettsia typhi | 0.0256 | 0.4609 |
| ROSCS | Roseiflexus castenholzii | 0.0725 | 0.4412 |
| ROSDO | Roseobacter denitrificans | 0.0776 | 0.4382 |
| ROSS1 | Roseiflexus sp | 0.0765 | 0.4469 |
| RUBXD | Rubrobacter xylanophilus | 0.0983 | 0.4179 |
| RUTMC | Ruthia magnifica subsp. Calyptogena magn | 0.0226 | 0.4165 |
| SACD2 | Saccharophagus degradans | 0.0674 | 0.4817 |
| SACEN | Saccharopolyspora erythraea | 0.1551 | 0.5023 |
| SALAI | Salinispora arenicola | 0.1403 | 0.4879 |
| SALAR | Salmonella arizonae | 0.0559 | 0.4320 |
| SALCH | Salmonella choleraesuis | 0.0574 | 0.4345 |
| SALPA | Salmonella paratyphi A | 0.0532 | 0.4263 |
| SALPB | Salmonella paratyphi B | 0.0564 | 0.4501 |
| SALRD | Salinibacter ruber | 0.2217 | 0.5266 |
| SALTI | Salmonella typhi | 0.0582 | 0.4385 |
| SALTO | Salinispora tropica | 0.1463 | 0.4951 |
| SALTY | Salmonella typhimurium | 0.0563 | 0.4205 |
| SERP5 | Serratia proteamaculans | 0.0556 | 0.4156 |
| SHEAM | Shewanella amazonensis | 0.0542 | 0.4284 |
| SHEB5 | Shewanella baltica | 0.0499 | 0.4197 |
| SHEB8 | Shewanella baltica | 0.0510 | 0.4229 |
| SHEB9 | Shewanella baltica | 0.0527 | 0.4315 |
| SHEDO | Shewanella denitrificans | 0.0498 | 0.4394 |
| SHEFN | Shewanella frigidimarina | 0.0467 | 0.4142 |
| SHEHH | Shewanella halifaxensis | 0.0471 | 0.4240 |
| SHELP | Shewanella loihica | 0.0574 | 0.4356 |
| SHEON | Shewanella oneidensis | 0.0491 | 0.4236 |
| SHEPA | Shewanella pealeana | 0.0489 | 0.4278 |
| SHEPC | Shewanella putrefaciens | 0.0455 | 0.4121 |
| SHESA | Shewanella sp | 0.0518 | 0.4247 |
| SHESH | Shewanella sediminis | 0.0528 | 0.4229 |
| SHESM | Shewanella sp | 0.0503 | 0.4212 |
| SHESR | Shewanella sp | 0.0518 | 0.4230 |
| SHESW | Shewanella sp | 0.0475 | 0.4119 |
| SHEWM | Shewanella woodyi | 0.0545 | 0.4292 |
| SHIB3 | Shigella boydii serotype 18 | 0.0563 | 0.4352 |
| SHIBS | Shigella boydii serotype 4 | 0.0560 | 0.4340 |
| SHIDS | Shigella dysenteriae serotype 1 | 0.0548 | 0.4295 |
| SHIF8 | Shigella flexneri serotype 5b | 0.0538 | 0.4282 |
| SHIFL | Shigella flexneri | 0.0543 | 0.4347 |
| SHISS | Shigella sonnei | 0.0557 | 0.4268 |
| SILPO | Silicibacter pomeroyi | 0.0757 | 0.4350 |
| SILST | Silicibacter sp | 0.0846 | 0.4507 |
| SINMW | Sinorhizobium medicae | 0.0713 | 0.4346 |
| SODGM | Sodalis glossinidius | 0.0672 | 0.4576 |
| SOLUE | Solibacter usitatus | 0.0769 | 0.4359 |
| SORC5 | Sorangium cellulosum | 0.1552 | 0.5289 |
| SPHAL | Sphingopyxis alaskensis | 0.0818 | 0.4334 |
| SPHWW | Sphingomonas wittichii | 0.0767 | 0.4012 |
| STAA1 | Staphylococcus aureus | 0.0817 | 0.4893 |
| STAA2 | Staphylococcus aureus | 0.0826 | 0.4888 |
| STAA3 | Staphylococcus aureus | 0.0815 | 0.4842 |
| STAA8 | Staphylococcus aureus | 0.0806 | 0.4872 |
| STAA9 | Staphylococcus aureus | 0.0826 | 0.4891 |
| STAAB | Staphylococcus aureus | 0.0654 | 0.4931 |
| STAAC | Staphylococcus aureus | 0.0835 | 0.4917 |
| STAAE | Staphylococcus aureus | 0.0814 | 0.4814 |
| STAAM | Staphylococcus aureus | 0.0817 | 0.4912 |
| STAAN | Staphylococcus aureus | 0.0820 | 0.4906 |
| STAAR | Staphylococcus aureus | 0.0755 | 0.4908 |
| STAAS | Staphylococcus aureus | 0.0710 | 0.4795 |
| STAAT | Staphylococcus aureus | 0.0779 | 0.4802 |
| STAAW | Staphylococcus aureus | 0.0802 | 0.4890 |
| STAEQ | Staphylococcus epidermidis | 0.0859 | 0.5010 |
| STAES | Staphylococcus epidermidis | 0.0858 | 0.4929 |
| STAHJ | Staphylococcus haemolyticus | 0.0853 | 0.4865 |
| STAS1 | Staphylococcus saprophyticus subsp. sapr | 0.0647 | 0.4723 |
| STRA1 | Streptococcus agalactiae serotype Ia | 0.0485 | 0.4486 |
| STRA3 | Streptococcus agalactiae serotype III | 0.0499 | 0.4447 |
| STRA5 | Streptococcus agalactiae serotype V | 0.0479 | 0.4571 |
| STRAW | Streptomyces avermitilis | 0.1705 | 0.5125 |
| STRCO | Streptomyces coelicolor | 0.1860 | 0.5229 |
| STRGC | Streptococcus gordonii | 0.0651 | 0.4742 |
| STRGG | Streptomyces griseus subsp. griseus | 0.1819 | 0.5166 |
| STRMK | Stenotrophomonas maltophilia | 0.0971 | 0.4507 |
| STRMU | Streptococcus mutans | 0.0411 | 0.4433 |
| STRP1 | Streptococcus pyogenes serotype M1 | 0.0498 | 0.4432 |
| STRP2 | Streptococcus pneumoniae serotype 2 | 0.0496 | 0.4541 |
| STRP3 | Streptococcus pyogenes serotype M3 | 0.0487 | 0.4477 |
| STRP6 | Streptococcus pyogenes serotype M6 | 0.0515 | 0.4359 |
| STRP8 | Streptococcus pyogenes serotype M18 | 0.0488 | 0.4284 |
| STRPB | Streptococcus pyogenes serotype M12 | 0.0519 | 0.4623 |
| STRPC | Streptococcus pyogenes serotype M12 | 0.0509 | 0.4630 |
| STRPD | Streptococcus pyogenes serotype M2 | 0.0527 | 0.4844 |
| STRPF | Streptococcus pyogenes serotype M4 | 0.0512 | 0.4784 |
| STRPG | Streptococcus pyogenes serotype M5 | 0.0473 | 0.4366 |
| STRPI | Streptococcus pneumoniae | 0.0499 | 0.4369 |
| STRPM | Streptococcus pyogenes serotype M28 | 0.0558 | 0.4764 |
| STRPN | Streptococcus pneumoniae | 0.0465 | 0.4544 |
| STRPS | Streptococcus pneumoniae | 0.0458 | 0.4539 |
| STRR6 | Streptococcus pneumoniae | 0.0487 | 0.4550 |
| STRS2 | Streptococcus suis | 0.0477 | 0.4674 |
| STRSV | Streptococcus sanguinis | 0.0655 | 0.4793 |
| STRSY | Streptococcus suis | 0.0484 | 0.4715 |
| STRT1 | Streptococcus thermophilus | 0.0422 | 0.4314 |
| STRT2 | Streptococcus thermophilus | 0.0434 | 0.4369 |
| STRTD | Streptococcus thermophilus | 0.0476 | 0.4553 |
| SULDN | Sulfurimonas denitrificans | 0.0264 | 0.4493 |
| SULMW | Sulcia muelleri | 0.0204 | 0.6778 |
| SULNB | Sulfurovum sp | 0.0334 | 0.4640 |
| SULSY | Sulfurihydrogenibium sp | 0.0186 | 0.4822 |
| SYMTH | Symbiobacterium thermophilum | 0.0859 | 0.4564 |
| SYNAS | Syntrophus aciditrophicus | 0.0484 | 0.4447 |
| SYNE7 | Synechococcus elongatus | 0.0597 | 0.4135 |
| SYNEL | Synechococcus elongatus | 0.0560 | 0.4434 |
| SYNFM | Syntrophobacter fumaroxidans | 0.0659 | 0.4761 |
| SYNJA | Synechococcus sp | 0.0760 | 0.4547 |
| SYNJB | Synechococcus sp | 0.0797 | 0.4615 |
| SYNP2 | Synechococcus sp | 0.0683 | 0.4817 |
| SYNP6 | Synechococcus sp | 0.0591 | 0.4111 |
| SYNPW | Synechococcus sp | 0.0944 | 0.4642 |
| SYNPX | Synechococcus sp | 0.0964 | 0.4543 |
| SYNR3 | Synechococcus sp | 0.0917 | 0.4506 |
| SYNS3 | Synechococcus sp | 0.0882 | 0.4617 |
| SYNS9 | Synechococcus sp | 0.0873 | 0.4604 |
| SYNSC | Synechococcus sp | 0.0965 | 0.4627 |
| SYNWW | Syntrophomonas wolfei subsp. wolfei | 0.0347 | 0.4434 |
| SYNY3 | Synechocystis sp | 0.0628 | 0.4642 |
| THEFY | Thermobifida fusca | 0.1443 | 0.5075 |
| THELT | Thermotoga lettingae | 0.0125 | 0.3474 |
| THEM4 | Thermosipho melanesiensis | 0.0111 | 0.4001 |
| THEMA | Thermotoga maritima | 0.0191 | 0.3889 |
| THEP1 | Thermotoga petrophila | 0.0178 | 0.3685 |
| THEP3 | Thermoanaerobacter pseudethanolicus | 0.0209 | 0.4110 |
| THEPX | Thermoanaerobacter sp | 0.0198 | 0.4205 |
| THESQ | Thermotoga sp | 0.0173 | 0.3630 |
| THET2 | Thermus thermophilus | 0.0495 | 0.4065 |
| THET8 | Thermus thermophilus | 0.0496 | 0.3853 |
| THETN | Thermoanaerobacter tengcongensis | 0.0186 | 0.4201 |
| THICR | Thiomicrospira crunogena | 0.0608 | 0.4293 |
| THIDA | Thiobacillus denitrificans | 0.0703 | 0.4271 |
| TREDE | Treponema denticola | 0.0341 | 0.5311 |
| TREPA | Treponema pallidum | 0.0521 | 0.4831 |
| TREPS | Treponema pallidum subsp. pallidum | 0.0521 | 0.4829 |
| TRIEI | Trichodesmium erythraeum | 0.0739 | 0.4796 |
| TROW8 | Tropheryma whipplei | 0.0553 | 0.4651 |
| TROWT | Tropheryma whipplei | 0.0549 | 0.4815 |
| UNCTG | Uncultured termite group 1 bacterium phy | 0.0204 | 0.4849 |
| UREP2 | Ureaplasma parvum serovar 3 | 0.0313 | 0.4993 |
| UREPA | Ureaplasma parvum | 0.0306 | 0.5066 |
| VEREI | Verminephrobacter eiseniae | 0.0863 | 0.4410 |
| VESOH | Vesicomyosocius okutanii subsp. Calyptog | 0.0210 | 0.4360 |
| VIBC3 | Vibrio cholerae serotype O1 | 0.0479 | 0.4161 |
| VIBCH | Vibrio cholerae | 0.0471 | 0.4232 |
| VIBF1 | Vibrio fischeri | 0.0494 | 0.4570 |
| VIBHB | Vibrio harveyi | 0.0594 | 0.4532 |
| VIBPA | Vibrio parahaemolyticus | 0.0550 | 0.4463 |
| VIBVU | Vibrio vulnificus | 0.0503 | 0.4260 |
| VIBVY | Vibrio vulnificus | 0.0499 | 0.4353 |
| WIGBR | Wigglesworthia glossinidia brevipalpis | 0.0138 | 0.6256 |
| WOLPM | Wolbachia pipientis wMel | 0.0541 | 0.5084 |
| WOLPP | Wolbachia pipientis | 0.0520 | 0.4827 |
| WOLSU | Wolinella succinogenes | 0.0297 | 0.4499 |
| WOLTR | Wolbachia sp. subsp. Brugia malayi | 0.0371 | 0.5080 |
| XANAC | Xanthomonas axonopodis pv. citri | 0.1029 | 0.4759 |
| XANC5 | Xanthomonas campestris pv. vesicatoria | 0.1051 | 0.4803 |
| XANC8 | Xanthomonas campestris pv. campestris | 0.0989 | 0.4668 |
| XANCB | Xanthomonas campestris pv. campestris | 0.1046 | 0.4806 |
| XANCP | Xanthomonas campestris pv. campestris | 0.0980 | 0.4633 |
| XANOM | Xanthomonas oryzae pv. oryzae | 0.1045 | 0.4923 |
| XANOP | Xanthomonas oryzae pv. oryzae | 0.1050 | 0.4898 |
| XANOR | Xanthomonas oryzae pv. oryzae | 0.1211 | 0.5129 |
| XANP2 | Xanthobacter autotrophicus | 0.0880 | 0.4551 |
| XYLF2 | Xylella fastidiosa | 0.0754 | 0.4450 |
| XYLFA | Xylella fastidiosa | 0.0840 | 0.4871 |
| XYLFM | Xylella fastidiosa | 0.0692 | 0.4581 |
| XYLFT | Xylella fastidiosa | 0.0739 | 0.4467 |
| YERE8 | Yersinia enterocolitica serotype O:8 / b | 0.0527 | 0.4100 |
| YERP3 | Yersinia pseudotuberculosis serotype O:1 | 0.0583 | 0.4152 |
| YERPA | Yersinia pestis bv. Antiqua | 0.0575 | 0.4079 |
| YERPB | Yersinia pseudotuberculosis serotype IB | 0.0565 | 0.4127 |
| YERPE | Yersinia pestis | 0.0562 | 0.4096 |
| YERPG | Yersinia pestis bv. Antiqua | 0.0571 | 0.4072 |
| YERPN | Yersinia pestis bv. Antiqua | 0.0562 | 0.4095 |
| YERPP | Yersinia pestis | 0.0574 | 0.4069 |
| YERPS | Yersinia pseudotuberculosis | 0.0575 | 0.4104 |
| YERPY | Yersinia pseudotuberculosis serotype O:3 | 0.0557 | 0.4066 |
| ZYMMO | Zymomonas mobilis | 0.0845 | 0.4546 |
