# Supplementary material for: Prediction of Protein Binding Regions in Disordered Proteins
Source: PLoS Comput Biol. 2009 May 1;5(5):e1000376. doi: 10.1371/journal.pcbi.1000376 (PMC2671142; doi:10.1371/journal.pcbi.1000376)
Supplement: Dataset S7 — The 44 complete eukaryota proteomes available from SwissProt (ftp://ftp.expasy.org/) used for full proteome scans. The fraction of total amino acids in disordered regions and the fraction of disordered amino acids in disordered binding sites are indicated together for each organism. (0.08 MB DOC) [file pcbi.1000376.s007.doc]

| SwissProt ID | Organism name | Fraction of amino acids in disordered regions | Fraction of disordered amino acids in binding regions |
| --- | --- | --- | --- |
| ANOGA | Anopheles gambiae | 0.2421 | 0.6103 |
| ARATH | Arabidopsis thaliana | 0.1675 | 0.5649 |
| ASHGO | Ashbya gossypii | 0.1840 | 0.5881 |
| ASPCL | Aspergillus clavatus | 0.2553 | 0.6169 |
| ASPFU | Aspergillus fumigatus | 0.2444 | 0.6146 |
| ASPNC | Aspergillus niger | 0.2261 | 0.6123 |
| ASPOR | Aspergillus oryzae | 0.2049 | 0.6015 |
| ASPTN | Aspergillus terreus | 0.2310 | 0.6170 |
| BOTFB | Botryotinia fuckeliana | 0.2689 | 0.6068 |
| CAEBR | Caenorhabditis briggsae | 0.2028 | 0.5716 |
| CAEEL | Caenorhabditis elegans | 0.1986 | 0.5849 |
| CANAL | Candida albicans | 0.2190 | 0.5825 |
| CANGA | Candida glabrata | 0.2021 | 0.5659 |
| CHAGB | Chaetomium globosum | 0.3047 | 0.6393 |
| COCIM | Coccidioides immitis | 0.2702 | 0.6237 |
| CRYHO | Cryptosporidium hominis | 0.1408 | 0.5376 |
| CRYNE | Cryptococcus neoformans | 0.3168 | 0.6231 |
| DEBHA | Debaryomyces hansenii | 0.1884 | 0.5688 |
| DICDI | Dictyostelium discoideum | 0.2423 | 0.5600 |
| DROME | Drosophila melanogaster | 0.3021 | 0.6197 |
| DROPS | Drosophila pseudoobscura | 0.2511 | 0.6233 |
| ENCCU | Encephalitozoon cuniculi | 0.0714 | 0.5025 |
| KLULA | Kluyveromyces lactis | 0.1814 | 0.5702 |
| LODEL | Lodderomyces elongisporus | 0.2469 | 0.5939 |
| MAGGR | Magnaporthe grisea | 0.2974 | 0.6341 |
| MONBE | Monosiga brevicollis | 0.2214 | 0.6343 |
| NEMVE | Nematostella vectensis | 0.1587 | 0.5691 |
| NEOFI | Neosartorya fischeri | 0.2396 | 0.6138 |
| NEUCR | Neurospora crassa | 0.3466 | 0.6380 |
| OSTLU | Ostreococcus lucimarinus | 0.1765 | 0.5165 |
| OSTTA | Ostreococcus tauri | 0.2053 | 0.5312 |
| PHANO | Phaeosphaeria nodorum | 0.2569 | 0.6167 |
| PICGU | Pichia guilliermondii | 0.1830 | 0.5713 |
| PICST | Pichia stipitis | 0.1698 | 0.5540 |
| PLAYO | Plasmodium yoelii yoelii | 0.1970 | 0.5886 |
| SCHPO | Schizosaccharomyces pombe | 0.1583 | 0.5390 |
| SCLS1 | Sclerotinia sclerotiorum | 0.2719 | 0.6044 |
| THEAN | Theileria annulata | 0.1277 | 0.5387 |
| THEPA | Theileria parva | 0.1354 | 0.5505 |
| USTMA | Ustilago maydis | 0.3384 | 0.6329 |
| VANPO | Vanderwaltozyma polyspora | 0.2074 | 0.5529 |
| YARLI | Yarrowia lipolytica | 0.2506 | 0.5966 |
| YEAS7 | Saccharomyces cerevisiae | 0.1967 | 0.5625 |
| YEAST | Saccharomyces cerevisiae | 0.2003 | 0.5642 |
