# Supplementary material for: Prediction of Protein Binding Regions in Disordered Proteins
Source: PLoS Comput Biol. 2009 May 1;5(5):e1000376. doi: 10.1371/journal.pcbi.1000376 (PMC2671142; doi:10.1371/journal.pcbi.1000376)
Supplement: Figure S1 — Development of ANCHOR. In the first step, our Short Disordered Binding Sites dataset and Globular Proteins dataset (positive and negative datasets) are split up and only 2/3 is used in the subsequential steps. Then a parameter set (w1, w2, p1, p2, p3) is selected from the 144,000 random ones. This parameter set is used to calculate S, Eint and Egain for every position in every sequence in the three input datasets using the fixed energy predictor matrix P (see Theory). Based on this calculations the evaluating measures are calculated: TPR is calculated on Short Disordered Binding Sites, FPR is calculated on Globular Proteins and F is calculated on Disordered Proteins. Based on these measures, the best parameter set out of 144,000 is chosen (see Data and Methods). Then this parameter set is evaluated on the remaining one third of the datasets. These results are reported in Table 3. This procedure is repeated for all three subsets of Short Disordered Binding Sites and Globular Proteins. The output of the three optimized predictors are combined into one final predictor by averaging their output. (0.05 MB PPT) [file pcbi.1000376.s008.ppt]

## Slide 1
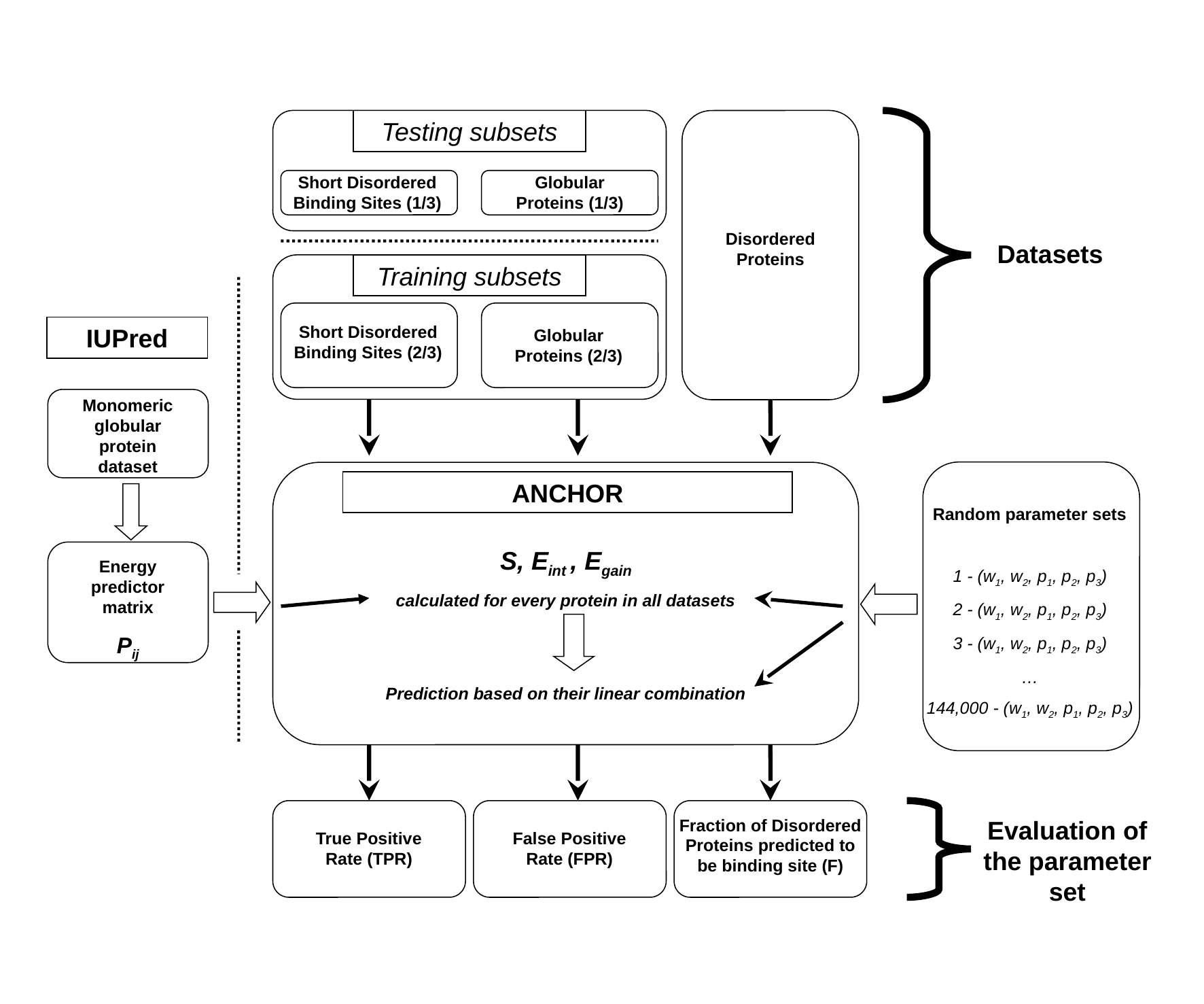

Testing subsets
Short Disordered Binding Sites (1/3)
Globular Proteins (1/3)
Disordered Proteins
Datasets
Training subsets
Short Disordered Binding Sites (2/3)
IUPred
Globular Proteins (2/3)
Monomeric globular protein dataset
ANCHOR
Random parameter sets
1 - (w1, w2, p1, p2, p3)
2 - (w1, w2, p1, p2, p3)
3 - (w1, w2, p1, p2, p3)
…
144,000 - (w1, w2, p1, p2, p3)
S, Eint , Egain
calculated for every protein in all datasets
Prediction based on their linear combination
Energy predictor matrix
Pij
Evaluation of the parameter set
Fraction of Disordered Proteins predicted to be binding site (F)
True Positive Rate (TPR)
False Positive Rate (FPR)
