# Supplementary figures and images for: Prediction of Protein Binding Regions in Disordered Proteins
Source: PLoS Comput Biol. 2009 May 1;5(5):e1000376. doi: 10.1371/journal.pcbi.1000376 (PMC2671142; doi:10.1371/journal.pcbi.1000376)

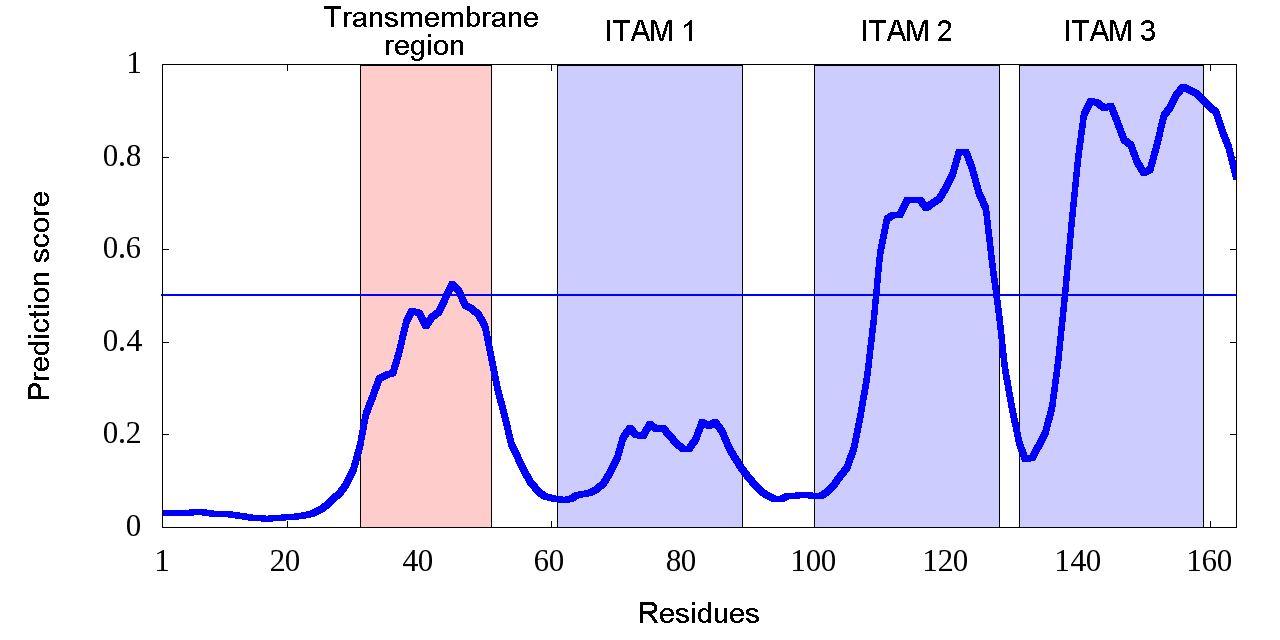

Supplement: Figure S6 — ANCHOR prediction output for the ζ-chain of T-cell receptor. Prediction output for the zeta-chain of the T-cell receptor. The transmembrane region is marked with red box and the three intracellular ITAM regions are marked with blue boxes. (0.12 MB TIF) [file pcbi.1000376.s013.tif]
