# Supplementary material for: Prediction of Protein Binding Regions in Disordered Proteins
Source: PLoS Comput Biol. 2009 May 1;5(5):e1000376. doi: 10.1371/journal.pcbi.1000376 (PMC2671142; doi:10.1371/journal.pcbi.1000376)
Supplement: Protocol S1 — Protocol including references for the Supporting Information. (0.04 MB DOC) [file pcbi.1000376.s014.doc]

# Supplementary Material

### Definition of secondary structure types

The secondary structure definitions of amino acids were generated with DSSP [1] considering only three groups: helical (H), extended (E) and coil (C). Based on this 7 types of protein interfaces can be defined taking into consideration the amount of each of the three basic secondary structural elements present in the particular interface (H, E or C or any combination of these three, for example H+C means that there is a substantial amount of interacting amino acids in both helical and coil conformation).

- ‘H’ attribute was assigned to an interface if more than 40% of its residues are in helical conformation and/or it contains at least 7 consecutive helical amino acids.
- ‘E’ attribute was assigned if more than 40% of the interacting residues are in extended conformation or any of the following patterns were found in the interface: [ET]EEE[ET], EE[ET]{0,3}EEE or EEE[ET]{0,3}EE, where T stands for turn conformation, [ET] denotes a residue in either E or T conformation and {n,m} means that the preceding amino acid is present at least n but at most m times in a row (eg. the pattern EETETEEE is matched by the second expression).
- ‘C’ attribute was assigned if at least half of the amino acids are in coil or turn conformation or there are at least 8 consecutive coil or turn residues or if neither ‘H’ nor ‘E’ could be assigned.

References for Supplementary Protocols, Figures and Tables:

1. Kabsch W, Sander C (1983) Dictionary of protein secondary structure: pattern recognition of hydrogen-bonded and geometrical features. Biopolymers 22: 2577-2637.

2. Dosztanyi Z, Csizmok V, Tompa P, Simon I (2005) The pairwise energy content estimated from amino acid composition discriminates between folded and intrinsically unstructured proteins. J Mol Biol 347: 827-839.

3. Meszaros B, Tompa P, Simon I, Dosztanyi Z (2007) Molecular principles of the interactions of disordered proteins. J Mol Biol 372: 549-561.

4. Oldfield CJ, Meng J, Yang JY, Yang MQ, Uversky VN, et al. (2008) Flexible nets: disorder and induced fit in the associations of p53 and 14-3-3 with their partners. BMC Genomics 9 Suppl 1: S1.

5. Sampietro J, Dahlberg CL, Cho US, Hinds TR, Kimelman D, et al. (2006) Crystal structure of a beta-catenin/BCL9/Tcf4 complex. Mol Cell 24: 293-300.

6. Kiss R, Kovács D, Tompa P, Perczel A (2008) Local structural preferences of calpastatin, the intrinsically unstructured protein inhibitor of calpain. Biochemistry 47: 6936-6945.

7. Radhakrishnan I, Perez-Alvarado GC, Parker D, Dyson HJ, Montminy MR, et al. (1997) Solution structure of the KIX domain of CBP bound to the transactivation domain of CREB: a model for activator:coactivator interactions. Cell 91: 741-752.

### 
